# Nematophin, an Antimicrobial Dipeptide Compound From *Xenorhabdus nematophila* YL001 as a Potent Biopesticide for *Rhizoctonia solani* Control

**DOI:** 10.3389/fmicb.2019.01765

**Published:** 2019-08-07

**Authors:** Shujing Zhang, Qi Liu, Yunfei Han, Jinghua Han, Zhiqiang Yan, Yonghong Wang, Xing Zhang

**Affiliations:** ^1^Key Laboratory of Plant Protection Resources and Pest Management of Ministry of Education, Research and Development Center of Biorational Pesticides, College of Plant Protection, Northwest A&F University, Yangling, China; ^2^Plant Quarantine and Protection Bureau of Zhumadian, Zhumadian, China

**Keywords:** *Xenorhabdus nematophila* YL001, dipeptides, nematophin, enantiomeric mixture, antimicrobial activity, sclerotial development

## Abstract

This study was conducted to purify and identify metabolites of antimicrobial activity against phytopathogens from *Xenorhabdus nematophila* YL001. Three dipeptide compounds were purified from its cell-free cultural broth and identified as (±)-nematophin, *cyclo* (L-Pro-Gly), and *N*, *N′*-dimethyl-*cyclo* (L-Phe-L-Leu). Nematophin demonstrated a wider antifungal spectrum than the other two compounds. It also exhibited strong inhibitory effects on mycelial growth of *Rhizoctonia solani* and *Phytophthora infestans* with EC_50_ values of 40.00 and 51.25 μg/ml, respectively. Its (*S*)-configuration structure [(+)-nematophin] was also synthesized and exhibited higher antimicrobial activity than the enantiomeric mixture. The detached leaf assay revealed that nematophin possessed significant preventive and curative efficacy against *R. solani* on broad bean leaves showing corresponding control efficacies of 93.01 and 94.93% at 1,000 μg/ml, comparable to those of a chemical fungicide (carbendazim) at 500 μg/ml. Additionally, the pot experiments indicated that nematophin could effectively inhibit the disease extension on rice and broad bean plants caused by *R. solani*. Nematophin also exerted some adverse influences on the sclerotial development of *R. solani* by dramatically suppressing their formation and maturation at 40.00 μg/ml, as well as their germination at 15.00 μg/ml. Morphological and ultrastructural observations showed that the hyphae of *R. solani* became twisted, shriveled, and deformed at the growing points after exposure to nematophin at 40.00 μg/ml, and that the subcellular fractions also became abnormal concurrently, especially the mitochondrial structure. These results indicate that nematophin has great potential to be used as a bio-pesticide in agricultural production.

## Introduction

*Xenorhabdus nematophila*, a well-studied genus of entomopathogenic bacteria, lives in symbiosis with *Steinernema nematodes*. Previous research has revealed that *X. nematophila* is a potent producer of natural compounds with versatile biological activities including antifungal, antibacterial, and antimalarial activities (Crawford et al., 2012; Challinor and Bode, 2015; Engel et al., 2017; Dreyer et al., 2018). Many of these metabolites belong to peptides such as xenocoumacins, nematophin, xenorhabdins, rhabduscins, and xenorxides (Li et al., 1997a,b; Ji et al., 2004; Böszörményi et al., 2009; Helge, 2011; Yang et al., 2011). Such peptides are of diverse structures and resultant multiple bioactivities including antimicrobial activity against plant pathogens (Li et al., 1997a; Yang et al., 2011; Dreyer et al., 2018). Besides, whole genome programs have shown that approximately 7.5% of genomic genes encode proteins involving the secondary metabolism biosynthesis in the strain *X. nematophila* ATCC19061, most of which encoded molecules are cryptic (Chaston et al., 2011). Given that the culture supernatants of many Xenorhabdus species exhibited potent antimicrobial activity against many plant pathogens (Fang et al., 2014; Hazir et al., 2016; Sharma et al., 2016), it is feasible to discover novel secondary metabolites of antifungal activity from *X. nematophila* as bio-pesticides for agricultural production.

Plant diseases caused by fungi are destructive attacks on crop production, generating enormous economic losses worldwide. *Rhizoctonia solani* is a necrotrophic fungal pathogen that infects more than 200 plant species worldwide, resulting in severe losses in crop yield (Lewis and Lumsden, 2001; Zheng et al., 2013; Mayo et al., 2015; Zhou et al., 2016). It can also produce sclerotia, a special resting structure surviving in soil for many years, which is the main source of infection in the disease cycle (Townsend and Willetts, 1954; Feng et al., 2016; Moni et al., 2016). Currently, the most common strategy for disease management is the application of chemical fungicides. Their overuse, however, has posed serious threats to human health, environmental safety and ecological balance (Nicolopoulou-Stamati et al., 2016; Rohr et al., 2017). Bio-pesticides are effective alternatives to the chemical fungicides to overcome their adverse impacts (Kumar, 2018; Lengai and Muthomi, 2018). Moreover, searching for bioactive compounds from microbes for control of the difficult-to-control plant pathogens is becoming a promising lead in the development of novel antimicrobial agents for agriculture production (Kim and Hwang, 2007; Mnif and Ghribi, 2015; Kanagaraj Muthu-Pandian et al., 2018).

Previous studies revealed that the cell-free culture of *X. nematophila* YL001 exhibited potent antimicrobial activity against some plant pathogens *in vitro* and *in vivo*, including *P. infestans* and *B. cinerea* (Guo et al., 2017; Zhang et al., 2018). However, no purified bioactive compounds were obtained from this bacterial strain, although many metabolites of novel structures and strong antimicrobial activity were purified and identified from strains of genus *Xenorhabdus* (Dreyer et al., 2018) including odilorhabdins (Pantel et al., 2018), cabanillasin (Houard et al., 2013), and bicornutin-A (Böszörményi et al., 2009). These studies mainly focused on their medical applications as antimicrobial agents. Currently, there is still limited information available about the antimicrobial potency of the metabolites of *X. nematophila* against phytopathogens (Li et al., 1997a; Ji et al., 2004; Lang et al., 2008; Gualtieri et al., 2009; Dreyer et al., 2018), especially the *in vivo* antimicrobial data (Yang et al., 2011; Zhou et al., 2016). In this paper, we tried to isolate and identify metabolites of antimicrobial potency against agricultural pathogenic fungi and oomycetes from *X. nematophila* YL001 and determine their antimicrobial activity *in vitro*. Subsequently, we evaluated the *in vivo* control efficacy of (±)-nematophin against *R. solani*. The effects of nematophin on the sclerotial development of *R. solani*, as well as on the hyphal morphology and ultrastructure, were also observed in this paper. The results revealed that *N*,*N*′-dimethyl-*cyclo* (L-Phe-L-Leu) possessed efficient antifungal activity against *Exserohilum turcicum*, and that nematophin could be used as a potential candidate for bio-pesticides.

## Materials and Methods

### Strains and Growth Conditions

*X. nematophila* YL001 was isolated from its nematode symbiont, *Steinernema* sp. YL001, which was obtained from the soybean rhizosphere soil of Yangling, China (E: 107°59′—108°08′; N: 34°14′—34°20′; Soil type: Lou soil; average annual temperature: 12.9°C; average annual sunshine hours: 2163.8 h; average annual rainfall: 635.1 mm). Its morphological and biochemical characterization was performed in our laboratory with results listed in Supplementary Tables S1, S2. Additionally, its species was also identified as *X. nematophila* by 16S rDNA amplification (GenBank number: EU124381; Supplementary Figure S1A) and the genome sequence (GenBank number: NZ_CP032329; Supplementary Figure S1B).

The tested strains of eight phytopathogenic fungi (*R. solani*, *E. turcicum*, *Fusarium graminearum*, *Verticillium dahlia*, *Botrytis cinerea*, *Sclerotinia sclerotiorum*, *Alternaria alternata*, and *Gaeumannomyces graminis*) and two fungal-like oomycetes (*Phytophthora infestans* and *Phytophthora capsici*) were collected from fields in different regions of China which were identified and preserved in the laboratory of the Research and Development Center of Biorational Pesticides, Northwest A&F University, Yangling, Shaanxi, China. The information of their collection places and host plants can be seen in Supplementary Table S3. Each fungal or oomycete strain was activated by growing on the petri dishes containing potato-dextrose agar (PDA) culture medium for 2 or 3 days at 26°C before use.

### Microbial Fermentation

A single colony of cells of *X. nematophila* YL001 was cultured in a 500-ml flask containing 200 ml of fresh Luria-Bertani medium (LB: 1.0% Bacto tryptone, 0.5% yeast extract, and 1% NaCl in water; pH 7.2) for 12 h at 28°C with shaking at 180 rev/min. Then, 3.5 L of the culture broth (OD_600_ = 0.8) was transferred as a seed into a 70-L fermenter (Eastbio, China) containing 35 L of TSB medium (per liter of distilled water: peptone from soymeal 3 g, peptone from casein 17 g, NaCl 5 g, glucose 2.5 g, and K_2_HPO_4_ 2.5 g; pH 7.2) and the system was incubated at 28°C with continuous agitation of 160 rpm and aeration of 0.25 v/v/min. After 48-h culture, the broth of *X. nematophila* YL001 was centrifuged (10,000 *g*, 20 min, 4°C) to remove the bacterial cells. Then, the cell-free broth was concentrated to 7 L on a rotary evaporator at 35°C and stored at 4°C for use.

### Isolation and Characterization of Dipeptide Compounds

The concentrated cell-free broth (7 L) was extracted with petroleum ether (4 × 7 L), chloroform (4 × 7 L), and ethyl acetate (4 × 7 L), consecutively. The petroleum ether and ethyl acetate layers were dried over anhydrous sodium sulfate, filtered, and evaporated by a rotary evaporator to give their respective residues that were subjected to further purification.

**Figure 1:**
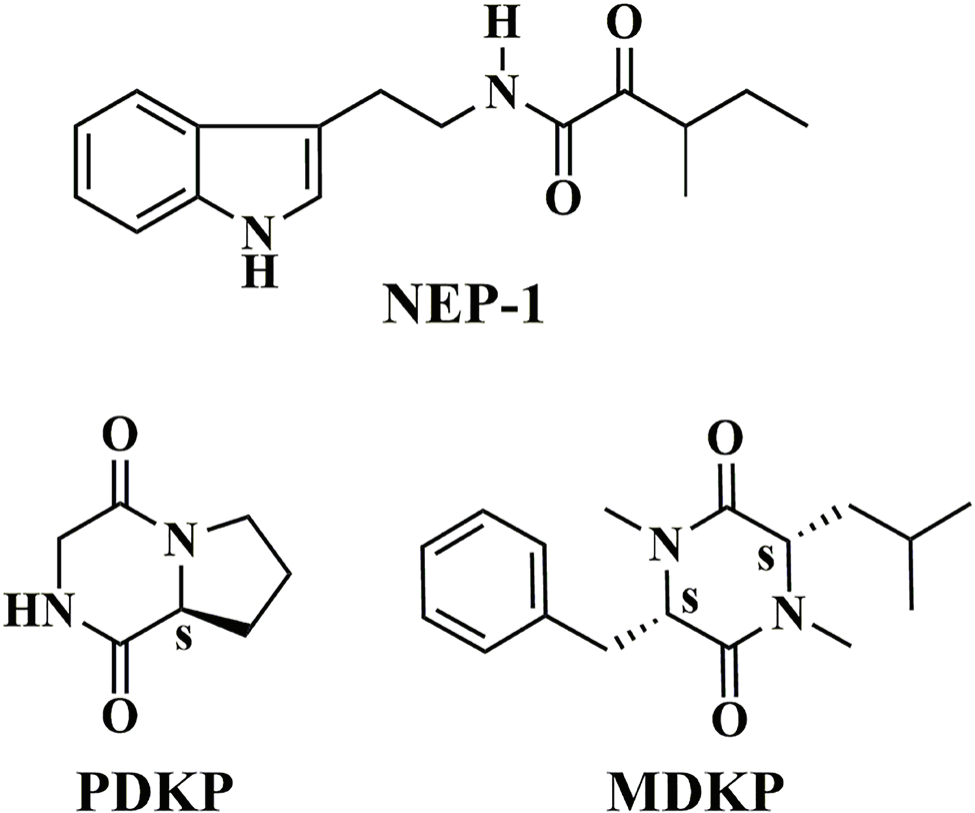
Chemical structures of **NEP-1**, **PDKP**, and **MDKP**.

The extract of petroleum ether (2.8 g) was then loaded onto a silica gel (90 g, 200–300 mesh, Qingdao Haiyang Chemical Co., Ltd., China) and eluted with a series of petroleum ether/ethyl acetate mixtures (1 L, v/v, 100:0, 75:1, 50:1, 30:1, 25:1, 15:1, 10:1, 5:1, 3:1, 2:1, 1:1, 0:1), sequentially. Each fraction was monitored by thin-layer chromatography (TLC). **NEP-1** was obtained from the eluent solutions of 25:1, 15:1, and 10:1, while **PDKP** was obtained from the eluent solutions of 5:1 and 3:1 (Figure 1).

The extract of ethyl acetate (4.1 g) was also purified by column chromatography on a silica gel (60 g, 200–300 mesh) using a series of chloroform/methanol mixtures (1 L, v/v, 50:1, 40:1, 20:1, 10:1, 5:1, 0:1) as the eluate. Each fraction was monitored by thin-layer chromatography (TLC). **MDKP** was obtained from the eluent solution of 100% methanol (Figure 1).

Structural identification of the three metabolites was performed on the basis of spectroscopic analysis. The mass spectra (MS) of the three compounds were obtained by a Mariner Mass 5,304 instrument (California, USA) or an AB Sciex TripleTOF 5,600 + System (Framingham, MA). ^1^H and ^13^C nuclear magnetic resonance (NMR) data were acquired on a Bruker AVANCE III 500 spectrometer (Rheinstetten, Germany) with tetramethylsilane as the internal standard. The optical rotation data were recorded on an Anton Paar MCP 300 polarimeter (Graz, Austria). The circular dichroism (CD) spectra were collected on an Applied Photophysics Chirascan spectropolarimeter (Leatherhead, UK).

**NEP-1**: ^1^H NMR (500 MHz, CDCl_3_) *δ* 8.08 (bs, 1H), 7.61 (dd, *J* = 7.8, 1.0 Hz, 1H), 7.38 (d, *J* = 8.1 Hz, 1H), 7.14 (td, *J* = 8.0, 7.0, 1.0 Hz, 1H), 7.14 (td, *J* = 8.0, 7.0, 1.0 Hz, 1H), 7.06 (bs, 1H), 7.04 (d, *J* = 2.3 Hz, 1H), 3.65 (q, *J* = 6.7 Hz, 2H), 3.50 (h, *J* = 6.9 Hz, 1H), 3.03 (t, *J* = 6.9 Hz, 2H), 1.80–1.67 (m, 1H), 1.50–1.34 (m, 1H), 1.09 (d, *J* = 7.0 Hz, 3H), 0.89 (t, *J* = 7.4 Hz, 3H); ^13^C NMR (125 MHz, CDCl_3_) *δ* 202.48, 160.18, 136.58, 127.29, 122.45, 122.14, 119.72, 118.78, 112.68, 111.40, 40.51, 39.66, 25.58, 25.31, 15.29, 11.62; ESI-MS (m/z): [M − H]^−^ calculated for C_16_H_19_N_2_O_2_ 271.14, found 271.10; HRESI-MS (m/z): [M + H]^+^ calculated for C_16_H_21_N_2_O_2_ 273.1603, found 273.1608; [M + Na]^+^ calculated for C_16_H_20_N_2_NaO_2_ 295.1422, found 295.1429.

**PDKP**: ${\left[\alpha \right]}_{D}^{25}$: −185.95 (c 0.83, EtOH); ^1^H NMR (500 MHz, D_2_O) *δ* 4.36 (t, *J* = 6.6 Hz, 1H), 4.21 (d, *J* = 17.3 Hz, 1H), 3.92 (d, *J* = 17.3 Hz, 1H), 3.65–3.48 (m, 2H), 2.47–2.29 (m, 1H), 2.47–2.29 (m, 1H), 2.05–1.86 (m, 2H); ^13^C NMR (125 MHz, D_2_O) *δ* 171.88, 165.96, 58.66, 45.52, 45.40, 27.84, 21.76; ESI-MS (m/z): [M + H]^+^ calculated for C_7_H_11_N_2_O_2_ 155.08, found 154.97; [M + Na]^+^ calculated for C_7_H_10_N_2_O_2_Na 177.06, found 176.97; HRESI-MS (m/z): [M + Na]^+^ calculated for C_7_H_10_N_2_O_2_Na 177.0640, found 177.0642.

**MDKP**: ${\left[\alpha \right]}_{D}^{25}$: −7.1 (c 0.38, CHCl_3_); ^1^H NMR (500 MHz, CDCl_3_) *δ* 7.38–7.25 (m, 1H), 7.17–7.12 (m, 1H), 4.21 (t, *J* = 4.7 Hz, 1H), 3.65 (dd, *J* = 9.2, 4.2 Hz, 1H), 3.33 (dd, *J* = 14.0, 4.9 Hz, 1H), 3.20 (dd, *J* = 14.0, 4.5 Hz, 1H), 2.98 (s, 3H), 2.90 (s, 3H), 1.75–1.68 (m, 1H), 0.87 (d, *J* = 6.5 Hz, 3H), 0.75 (d, *J* = 6.7 Hz, 3H), 0.70 (ddd, *J* = 13.7, 9.3, 4.2 Hz, 1H), 0.32 (ddd, *J* = 14.2, 9.2, 5.2 Hz, 1H); ^13^C NMR (125 MHz, CDCl_3_) *δ* 166.57, 165.19, 135.73, 130.02, 128.88, 127.60, 64.02, 60.06, 42.46, 37.72, 32.81, 32.55, 25.14, 22.71, 21.48; HRESI-MS (m/z): [M + H]^+^ calculated for C_17_H_25_N_2_O_2_ 289.1916, found 289.1907.

### Synthesis of an Enantiomeric Mixture of Nematophin (NEP-2) and Its (*S*)-Configuration Structure (NEP-3)

**Figure 2:**
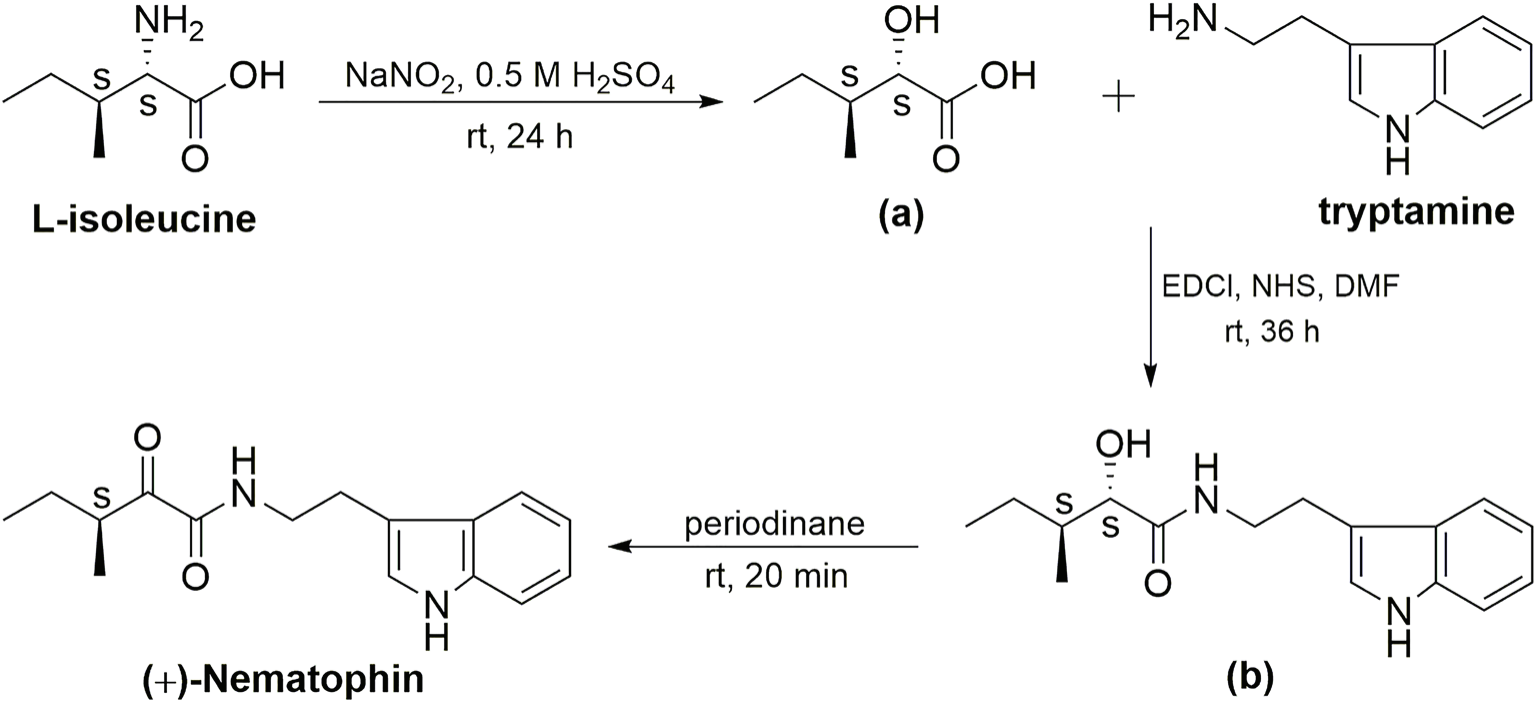
Synthetic route to the title compound (+)-nematophin (**NEP-3**).

(±)-Nematophin (**NEP-2**) was readily synthesized by one-pot amidation of (±)-2-keto-3-methylvaleric acid [(±)-KMVA] and tryptamine using ethyl-dimethylaminopropyl-carbodiimide hydrochloride (EDCl) as the condensation agent and *N*, *N*-dimethyl-4-aminopyridine (DMAP) as the catalyzer (Supplementary Scheme S1).

(+)-Nematophin (**NEP-3**) was also prepared by three reaction steps (Figure 2) according to a previously reported method with some modifications (Paik et al., 2003). L-Isoleucine [(2*S*, 3*S*)-isoleucine] was used as the chiral template material to prepare (2*S*, 3*S*)-hydroxypentanoic acid (**a**) by diazotization and hydrolysis in 0.5 M H_2_SO_4_ solution. Then, coupling of compound **a** with tryptamine was performed by a one-pot amidation method (EDCl/NHS) to give (2′*S*, 3′*S*)-N-(indol-3-ylethyl)-2′-hydroxy-3′-methylpentanamide (**b**). Finally, the title compound (3′*S*)-N-[2-(1H-Indol-3-yl) ethyl]-3-methyl-2-oxopentanamide [(+)-nematophin] was obtained by oxidation of **b** with Dess-Martin reagent (periodinane) in dichloromethane.

All the chemicals used were purchased from Aladdin Co. Ltd. (Beijing, China). The detailed synthetic procedures can be seen in Supplementary Material. The synthetic intermediates and target compounds were characterized identically to the reported structures by ^1^H NMR, ^13^C NMR, and ESI-MS (Supplementary Figures S16–S28).

### Effects of Three Isolated Compounds on Mycelial Growth of the Phytopathogenic Fungi and Oomycetes *in vitro*

The antimicrobial activity of three isolated compounds against the mycelial growth of 10 plant pathogens was performed as described in previous research with slight modifications (Zhang et al., 2018). Briefly, the three compounds were dissolved in dimethyl sulfoxide (DMSO) to prepare their respective stock solutions with a concentration of 10,000 μg/ml. A stock solution (150 μl) was mixed with 15 ml of molten PDA medium at a low temperature (about 40°C) to make a final drug concentration of 100 μg/ml (1% DMSO), and then the mixture was immediately poured into a petri dish (60-mm diameter) to form a plate of 2–3 mm thickness. PDA supplemented with the same volume of DMSO (1%) served as the blank control. Carbendazim (**MBC**) and mancozeb (**MZ**) at a concentration of 0.5 μg/ml were used as positive controls. After cooling, a 5-mm diameter mycelial disc was placed in the center of each plate with the inoculum side down. The plates were incubated at 28°C in dark until the mycelia reached the edges of the control dishes. Each experiment was repeated three times. The mycelia growth inhibition rate was calculated using the following formula:

$$\mathrm{Inhibition}\;\mathrm{rate}\;\left(\%\right)=\left[\left(D_{c}-D_{t}\right)/\left(D_{c}-0.5\right)\right]\times 100$$

where *D*_c_ and *D*_t_ represent the mycelial growth diameter of the control and treatment group, respectively. The diameter of the plug is 0.5 cm.

The antimicrobial activity of **NEP-2** and **-3** against five plant pathogens (*R. solani*, *S. sclerotiorum*, *P. infestans*, *F. graminearum*, and *P. capsici*) was also evaluated using the method as described above. The final concentration of **NEP-2** or -**3** in each test medium was 100 μg/ml and PDA plates supplemented with 1% DMSO were used as the controls.

The half-maximal effective concentration (effective dose for 50% inhibition, EC_50_) was derived from analysis of the concentration-dependent inhibition rates. PDA plates with serial concentrations of **NEP-1** (2.5, 5, 10, 20, 40, 60, 80, and 100 μg/ml) were used for test by the method described above. All experiments were independently performed three times under the same conditions.

### Antimicrobial Activity Assay of NEP-1 Against *R. solani* and *P. infestans* by a Detached Leaf Method

The efficacies of **NEP-1** on broad bean leaves infected with *R. solani* and potato leaves infected with *P. infestans* were evaluated according to the studies (Arfaoui et al., 2018) with some modifications. Broad bean (*Vicia faba* L.) was grown in a growth chamber (25 ± 1°C, 75 ± 10% RH, and a 12:12 LD photoperiod) for 21 days. Potato tuber was grown for 30 days under the same conditions. **NEP-1** solutions of 500 and 1,000 μg/ml were prepared by dissolving it in an aqueous solution (sterile water composed of 0.5% tween 20, v/v). **MBC** solution of 500 μg/ml in the same mixture was used as the positive control against *R. solani* and **MZ** solution of 500 μg/ml against *P. infestans*. Sterile water containing 0.5% tween 20 was used as the blank control. All experiments were arranged in a plant growth chamber (25 ± 1°C, 75 ± 10% RH, and a 12:12 LD photoperiod) with nine leaves per treatment and were repeated three times.

For the protective activity assay, nine leaves of broad bean or potato were as a group and sprayed with a pre-prepared sample solution (1 ml for each leaf). After incubation for 12 h, a 5-mm diameter mycelial disc was inoculated onto the center of each leaf that was wounded at the inoculation site using a sterilized needle (avoiding the main vein of the leaves) in advance. After incubation for another 2 days, the lesion area was quantified from the captured images with Image J 1.38x software^1^, and the control efficacy was also calculated according to the following formula:

$$\mathrm{Control}\;\mathrm{efficacy}\;\left(\%\right)=\left[\left(A_{c}-A_{t}\right)/\left(A_{c}-0.25\right)\right]\times 100$$

where *A*_c_ and *A*_t_ represent the disease area of the blank control and treatment group, respectively. The area of the mycelial disc is 0.25 cm^2^.

For the curative activity assay, nine leaves of broad bean or potato were as a group and each leaf was inoculated with a diameter mycelial disc as described above. After incubation for 12 h (~3 mm lesion around each disc), nine leaves of a group were sprayed with a pre-prepared sample solution of **NEP-1** (500 or 1,000 μg/ml), **MBC** (500 μg/ml) or **MZ** (500 μg/ml) (1 ml for each leaf). The subsequent operations were as described above.

### Biocontrol Efficacies of NEP-1 Against *R. solani* on Rice and Broad Bean Plants Grown in a Greenhouse

A pot experiment was performed to assess the *in vivo* biocontrol efficiency of **NEP-1** against *R. solani*. Potted rice plants (35 days after seeding) and broad bean plants (30 days after seeding) grown in a greenhouse were used as the host plants. The temperature of the greenhouse ranged from 28°C (±2°C) in the daytime to 22°C (±2°C) during nighttime, and the relative humidity was controlled at 85% (±5%). The procedures of fungal inoculum, pathogen inoculation, and disease evaluation were according to the reported literature (Park et al., 2008; Wu et al., 2019) with some modifications. Briefly, 10 agar blocks (0.5-cm diameter) from the outer edge of a 3-day-old inoculated PDA plate were inoculated in a 250-ml Erlenmeyer flask with 100 ml of PDB (potato dextrose broth) medium. After incubation on a shaker (160 rpm) under darkness for 7 days, liquid cultured mycelia were harvested and cut into small mycelial balls (0.1 g in wet weight). A mycelial ball was placed beneath the leaf sheath and covered with aluminum foil immediately. When typical lesions appeared after 3 days, the aluminum foil was removed. Then the plants of a group were sprayed with a sample solution of **NEP-1** (500 or 1,000 μg/ml), **MBC** (500 μg/ml), or sterile water. Three pots with more than five rice plants of each and nine pots with one broad bean plant of each were used as a group, respectively. Each experiment was performed with three replications. After 4-day incubation, the lesion length in each stem of the inoculated plants was recorded.

### Effects of NEP-1 on the Sclerotial Development of *R. solani*

The effects of **NEP-1** on sclerotial formation were performed according to a previously published procedure (Soltani et al., 2017). Briefly, serial concentrations of **NEP-1** in DMSO were added to PDA medium at final concentrations of 15, 20, 30, and 40 μg/ml, respectively. PDA supplemented with DMSO (1%) served as the control. A 5-mm diameter plug of *R. solani* was inoculated onto the center of each plate maintained at 28°C for 6 days until sclerotia manifested in the control plates. Additionally, the dynamic developmental process of sclerotia treated with 40 μg/ml of **NEP-1** was also observed at fixed time intervals of 3, 4, 6, and 12 days. All plates were photographed with a Nikon D500 camera (Tokyo, Japan), and the sclerotia phenotypes were observed by a LECIA M165 FC stereoscopic microscope (Leica-ULTRACUT, Wetzlar, Germany).

The effects of **NEP-1** on the sclerotial germination were measured according to a previously described method (Kazempour, 2004; Soltani et al., 2017). Uniform and healthy sclerotia were collected from PDA plates and soaked in solutions of various concentrations of **NEP-1** (15, 20, and 30 μg/ml) with 1% DMSO for 5 min. Then, the sclerotia treated with **NEP-1** at a certain concentration were placed onto PDA plates (one sclerotia per plate) and maintained at 28°C to observe their germination. The sclerotia treated with 1% DMSO were used as the controls. Each concentration was tested with 12 sclerotia and each test was repeated three times.

### Effects of NEP-1 on Hyphal Morphology and Ultrastructure of *R. solani*

Determination of the effects of **NEP-1** on the hyphal morphology and ultrastructure was conducted according to the method of Soner Soylu (Soylu et al., 2010). A prepared mycelial agar disc from a 2-day-old culture was inoculated in the center of the PDA plate with 40.00 μg/ml (EC_50_) of **NEP-1**, and the plate was incubated at 28°C for 2 days in the dark. PDA plates without **NEP-1** treatment were used as the controls.

For the scanning electron microscopy (SEM) observations, mycelial discs (2 mm × 4 mm × 4 mm) were fixed with 2.5% glutaraldehyde in a phosphate buffer (0.1 M, pH = 7.2) overnight at 4°C. Each sample was washed three times (20 min each) with 0.1 M phosphate buffer (pH = 7.2) to remove the excess glutaraldehyde. After fixation, the samples were dehydrated in a graded ethanol series (twice at 30, 50, 70, 80, and 90% and three times at 100%, v/v) for 20 min in each solution. After dehydration, the samples were dipped into isoamylacetate three times (20 min each) for replacement of ethanol. Finally, each sample was dried at the critical point using supercritical carbon dioxide. All samples were fixed on a holder using double-sided tape and coated with gold using an E1010 sputter coating machine (Hitachi, Tokyo, Japan) for 90 s at 9 mA. Samples were then imaged using a JSM-6360LV SEM instrument (JEOL, Tokyo, Japan).

For the transmission electron microscopy (TEM) observations, the pre-fixed mycelia were the same as described in the SEM method. After washing with a phosphate buffer (0.1 M, pH = 7.2) three times, the samples were post-fixed with 1% osmic acid for 2 h. Then, the samples were washed again with the phosphate buffer (0.1 M, pH 7.2) immediately followed by dehydration as described in the SEM method. Samples were dipped into ethanol twice (30 min each), after which the specimens were passed through a solution of epoxy resin/epoxy propane (1:1, v/v) for 1 h and embedded in epoxy media at 55°C for 48 h. Blocks were sectioned using a diamond knife with an Ultramicrotome (Leica-ULTRACUT, Wetzlar, Germany) into ultrathin sections of approximately 70 nm. The ultrathin sections were contrasted with 2% uranyl acetate and 2% lead citrate for 30 min prior to examination on a JEM-1230 TEM (Hitachi, Tokyo, Japan). At least three samples from each of the treated and control groups were examined by SEM and TEM.

### Data Processing and Analysis

All datasets were analyzed by ANOVA using Statistica Software (Statsoft, Tulsa, OK, USA). The EC_50_ was calculated by linear regression of the log of the colony diameter versus the various **NEP-1** concentrations. When the ANOVA was significant, means were separated with the least significant difference test (LSD, *p* < 0.05).

## Results

### Structure Identification

In the present study, three metabolites (Figure 1) were isolated from the fermented supernatant of *X. nematophila* YL001. Their structures were characterized by ^1^H NMR, ^13^C NMR, and MS spectra. According to the relevant spectral data reported, the three compounds were identified as nematophin (**NEP-1**, Supplementary Figures S5–S8) (Li et al., 1997a), hexahydropyrrolo[1, 2-α]pyrazine-1,4-dione (**PDKP**, Supplementary Figures S9–S12) (Bishay et al., 2018) and 1,4-dimethyl-3-(2-methylpropyl)-6-(phenylmethyl)-2,5-piperazinedione (**MDKP**, Supplementary Figures S13–S15) (Nakao et al., 2016), respectively. They are dipeptide compounds in structure. Referring to the existing optical rotation data, the absolute stereostructures of **PDKP** and **MDKP** were determined as (8α*S*)-hexahydropyrrolo[1, 2-α]pyrazine-1,4-dione [*cyclo*(L-Pro-Gly)] (Campbell et al., 2009) and (3*S*, 6*S*)-1,4-dimethyl-3-(2-methylpropyl)-6-(phenylmethyl)-2,5-piperazinedione [*N*, *N*′-dimethyl-*cyclo*(L-Phe-L-Leu)] (Nakao et al., 2016), respectively.

To confirm the stereo structure of **NEP-1**, an enantiomeric mixture of nematophin (**NEP-2**, Supplementary Figures S16–S19) and its (*S*)-configuration structure (**NEP-3**, Supplementary Figures S25–S28) were synthesized and characterized in this study. Their circular dichroism (CD) spectra were recorded with the results shown in Supplementary Figure S2. An obvious positive absorption band was observed from 300 to 400 nm for the **NEP-3** solution. Interestingly, no absorption signals were detected in this wavelength range for both **NEP-1** and **NEP-2** solutions. Moreover, different from **NEP-1** and **NEP-2** of racemic property, **NEP-3** also exhibited a specific rotation ${\left[\alpha \right]}_{D}^{25}$ value of 31.66 (c 0.58) in CHCl_3_. These findings indicate that **NEP-1** is an enantiomeric mixture.

### Examination of Inhibitory Effects of Three Isolated Compounds on Mycelia Growth of Agricultural Pathogenic Fungi and Oomycetes *in vitro*

A previous study revealed that **NEP-1** had strong *in vitro* bioactivity against a series of fungal and bacterial species (Li et al., 1997a). Piperazine-2,5-diones including **PDKP** and **MDKP** are an important class of cyclodipeptide compounds of versatile bioactivities (Borthwick, 2012). However, scarce work described their antimicrobial activity against plant pathogens. Thus, to further explore their potential as bio-pesticides, we evaluated their inhibitory effects on mycelial growth of 10 common phytopathogens *in vitro* with results listed in Table 1. **NEP-1** showed a broad antimicrobial spectrum and exhibited high inhibitory effects against *R. solani*, *P. infestans*, and *F. graminearum*, with inhibition rates of 82.74, 80.41, and 80.93%, respectively. *R. solani* was most sensitive to **NEP-1**. Moreover, the EC_50_ values of **NEP-1** against *R. solani* and *P. infestans* were determined to be 40 and 51.25 μg/ml, respectively (Table 2). Noteworthily, **NEP-1** also exhibited higher inhibition rates at 100 μg/ml than **MBC** at 0.5 μg/ml against the selected pathogens except *F. graminearum*, *P. capsica*, and *S. sclerotiorum*. Similarly, compared to the antimicrobial activity of **MZ** at 0.5 μg/ml, higher inhibition rates were observed for **NEP-1** at 100 μg/ml against the tested pathogens except *E. turcicum* and *G. gramini*. **PDKP** displayed a moderate inhibitory effect against *G. graminis* (inhibition rate of 35%) and weak inhibitory effects against *R. solani*, *P. infestans*, *S. sclerotiorum*, and *A. alternate* (inhibition rate < 40%) at 100 μg/ml. **MDKP**, however, remarkably inhibited the mycelial growth of *E. turcicum* with an inhibition rate of 67.60% at 100 μg/ml, higher than that of **NEP-1**. These results indicate that **NEP-1** is an excellent antifungal agent with a broad spectrum and that **MDKP** has the potential to control the maize leaf spot disease caused by *E. turcicum*.

**Table 1:** Inhibitory effects of the three isolated compounds on the mycelial growth of 10 plant pathogens.

Pathogenic fungi	Inhibition rate (%)				
	NEP-1	PDKP	MDKP	MBC	MZ
*Rhizoctonia solani*	82.74 ± 0.29a	14.08 ± 0.05	<5.00	70.70 ± 0.15^*^	24.39 ± 0.10^*^
*Exserohilum turcicum*	63.78 ± 0.33e	5.86 ± 0.09	67.60 ± 0.11^*^	<5.00	79.38 ± 0.08^*^
*Phytophthora infestans*	80.41 ± 0.16b	14.24 ± 0.32	<5.00	8.84 ± 0.47^*^	25.42 ± 0.34^*^
*Fusarium graminearum*	80.93 ± 0.08b	6.74 ± 0.05	<5.00	92.78 ± 0.08^*^	21.44 ± 0.50^*^
*Verticillium dahliae*	75.73 ± 0.05c	<5.00	<5.00	18.96 ± 0.12^*^	13.79 ± 0.05^*^
*Phytophthora capsici*	68.42 ± 0.13d	<5.00	<5.00	81.59 ± 0.10^*^	< 5.00
*Botrytis cinerea*	65.06 ± 0.10e	<5.00	24.35 ± 0.04	14.88 ± 0.28^*^	25.88 ± 0.05^*^
*Sclerotinia sclerotiorum*	61.95 ± 0.09f	18.48 ± 0.04	<5.00	99.17 ± 0.06^*^	<5.00
*Alternaria alternate*	59.23 ± 0.08f	8.12 ± 0.24	<5.00	<5.00	15.36 ± 0.05^*^
*Gaeumannomyces gramini*	49.75 ± 0.10g	35.00 ± 0.05	<5.00	46.05 ± 0.12^*^	73.73 ± 0.10^*^

*The concentration of **NEP-1**, **PDKP**, or **MDKP** was set as 100 μg/ml. Carbendazim (**MBC**) and mancozeb (**MZ**) at a concentration of 0.5 μg/ml were used as positive controls. Different lowercase letters in the same column indicate significant differences at p < 0.05. The asterisk indicates significant difference exists as compared to **NEP-1** (p < 0.05, Student’s t test). Data are presented as the average ± the standard deviation for three replicates*.

**Table 2:** Inhibitory effects of nematophin against *R. solani* and *P. infestans*.

Strain	Regression curve	EC_50_^a^ (CI_95_^b^) (μg/ml)	*χ*^c^
*R. solani*	*y* = 4.12*x* + 1.31	40.00 (37–48)	3.61
*P. infestans*	*y* = 4.85*x* − 8.29	51.25 (45–60)	0.52

a *Effective dose for 50% inhibition as compared with the control*.

b *95% confidence intervals*.

c *χ^2^ value, significant at p < 0.05 level*.

To explore whether the configuration of nematophin affects its antimicrobial activity, we tested the inhibition of three nematophin compounds (**NEP-1**, **-2**, and **-3**) on mycelia growth of five plant pathogens. As revealed in Table 3, compared to **NEP-1** and **-2**, **NEP-3** exhibited stronger inhibition against the five selected pathogens and significantly higher inhibition rates against *S. sclerotiorum* and *P. capsica*. Besides, no obvious differences were observed between **NEP-1** and **-2** in the inhibition rates. These results reveal that the (*S*)-configuration structure may possess higher antimicrobial activity than the (*R*)-enantiomer of nematophin and that **NEP-1** is an enantiomeric mixture. Given that **NEP-1** can be easily obtained through the bacterial fermentation of low cost, it was selected as the subject for subsequent experiments.

**Table 3:** Inhibitory effects of three nematophin compounds of decided configuration on the mycelial growth of five plant pathogens.

Pathogenic fungi	Inhibition rate^a^ (%)		
	NEP-1	NEP-2	NEP-3
*Rhizoctonia solani*	80.38 ± 0.24a	81.07 ± 0.18a	85.24 ± 0.34a
*Sclerotinia sclerotiorum*	64.29 ± 0.20b	60.54 ± 0.19b	71.68 ± 0.29a
*Phytophthora infestans*	78.02 ± 0.37ab	79.62 ± 0.24aa	85.13 ± 0.15^a^
*Fusarium graminearum*	80.57 ± 0.12a	79.94 ± 0.06a	85.35 ± 0.25a
*Phytophthora capsici*	64.02 ± 0.11b	68.62 ± 0.14b	75.13 ± 0.15a

a *The inhibitory rates of three compounds (100 μg/ml) on the mycelial growth of the pathogens*.

*Data are presented as the average ± the standard deviation for three replicates*.

*Different letters in each line*.

### Examination the Control Efficacies of NEP-1 Against *R. solani* and *P. infestans* on Their Respective Host Plant Leaves

**Figure 3:**
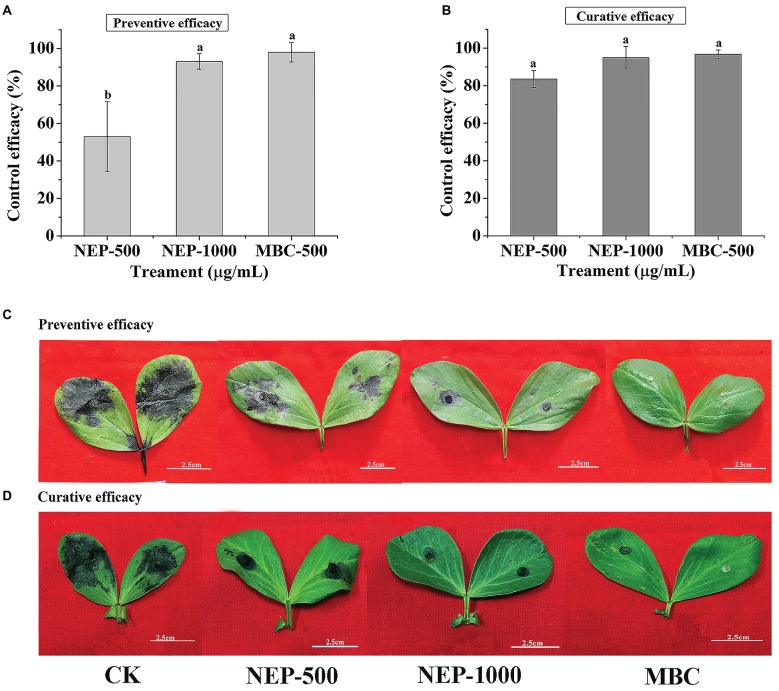
*In vivo* antifungal activity of **NEP-1** and carbendazim against *R. solani*. **(A,C)** Protective activity of **NEP-1** and **(B,D)** curative activity of **NEP-1**. Preventive/curative efficacy: the control efficacy of a compound that was sprayed on broad bean leaves 12 h before/after inoculation with *R. solani*. NEP500, 500 μg/ml of **NEP-1**; NEP1000, 1,000 μg/ml of **NEP-1**; **MBC**, 500 μg/ml of carbendazim.

As described above, **NEP-1** exhibited strong *in vitro* antimicrobial activity against *R. solani* and *P. infestans*. However, the antimicrobial activity *in vivo* was scarcely reported in previous studies. Thus, we explored the control efficacy of **NEP-1** against *R. solani* on broad bean leaves and against *P. infestans* on potato leaves. As shown in Figures 3A,C, **NEP-1** could protect the bean leaves from the infection of *R. solani* effectively, with preventive efficacies of 53.05% at 500 μg/ml and 93.01% at 1,000 μg/ml, which is comparable to that of the **MBC** treatment at 500 μg/ml (98.01%). Moreover, **NEP-1** also exhibited high curative activity for the diseased leaves infected by *R. solani*, with control efficacies of 83.59 and 94.93% at 500 and 1,000 μg/ml, respectively (Figures 3B,D).

The control efficacy of **NEP-1** against *P. infestans* on potato leaves is shown in Supplementary Figure S3. The results showed that **NEP-1** possessed weak preventive efficacy, with control efficacies of 15.58% at 500 μg/ml and 36.56% at 1,000 μg/ml (Supplementary Figures S3A,C), lower than that of **MZ** treatment at 500 μg/ml. Additionally, **NEP-1** exhibited moderate curative activity with control efficacies of 39.63% at 500 μg/ml and 56.41% and 1,000 μg/ml, lower than that of referenced **MZ** at 500 μg/ml (Supplementary Figures S3B,D). Overall, the disease development control assay indicated that **NEP-1** exhibited high control efficacy against *R. solani in vivo*. Therefore, *R. solani* was selected as the target pathogen for further experiments.

### Biocontrol Efficacies of NEP-1 Against *R. solani* on Rice and Broad Bean Plants

**Figure 4:**
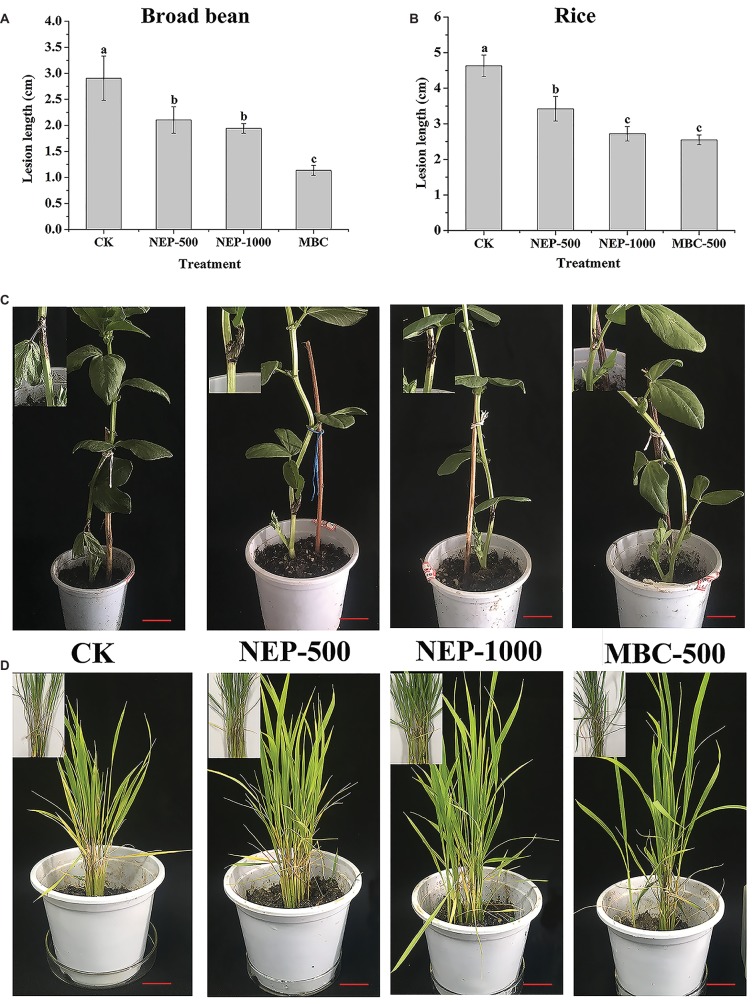
The lesion length and morphology of the plants infected by *R. solani* in pot experiments. **(A)** The lesion length of the broad bean plants; **(B)** the lesion length of the rice plants; **(C)** the morphology of the broad bean plants; and **(D)** the morphology of the rice plants. Insert: the disease area of the host plants. NEP500, 500 μg/ml of **NEP-1**; NEP1000, 1,000 μg/ml of **NEP-1**; **MBC**, 500 μg/ml of carbendazim; and CK was the blank control. Scale bar: 2.5 cm.

To further explore the potency of **NEP-1** as a new biopesticide, we evaluated the biocontrol potential of **NEP-1** against the infestation of *R. solani* on rice and broad bean plants using a pot experiment. The morphological variations as well as the average lesion length of the inoculated plants in different treatment groups are illustrated in Figure 4. In the blank control groups, both of the rice and broad bean plants were severely infected by *R. solani*, with an apparent symptom of sheath blight disease (Figures 4C,D). However, the disease severity was effectively alleviated in the **NEP-1** treatment groups (Figures 4C,D). Correspondingly, the lesion length on the inoculated plants in each **NEP-1** treatment group was much smaller than that in the corresponding blank control group (Figures 4A,B). Moreover, no obvious difference was observed in the lesion length on the rice plants between the **NEP-1** treatment group (1,000 μg/ml) and the **MBC** treatment group (500 μg/ml) (Figure 4B). These results indicated that **NEP-1** had a favorable biocontrol efficacy against the disease extension on rice and broad bean plants caused by *R. solani*.

### Effects of NEP-1 on the Sclerotial Development of *R. solani*

**Figure 5:**
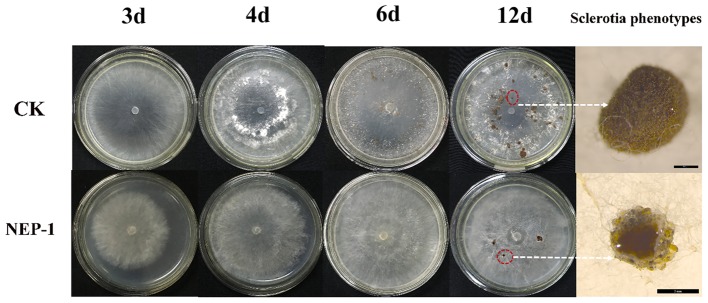
Effects of **NEP-1** on the sclerotial development of *R. solani*. CK, Control plate; **NEP-1**, plate treated with **NEP-1** at 40.00 μg/ml; plates were photographed at different incubating times (3, 4, 6, and 12 days). Sclerotia phenotypes were observed by a stereoscopic microscope after 12-day incubation. Scale bar: CK, 500 μm; **NEP-1**, 2 mm.

**Figure 6:**
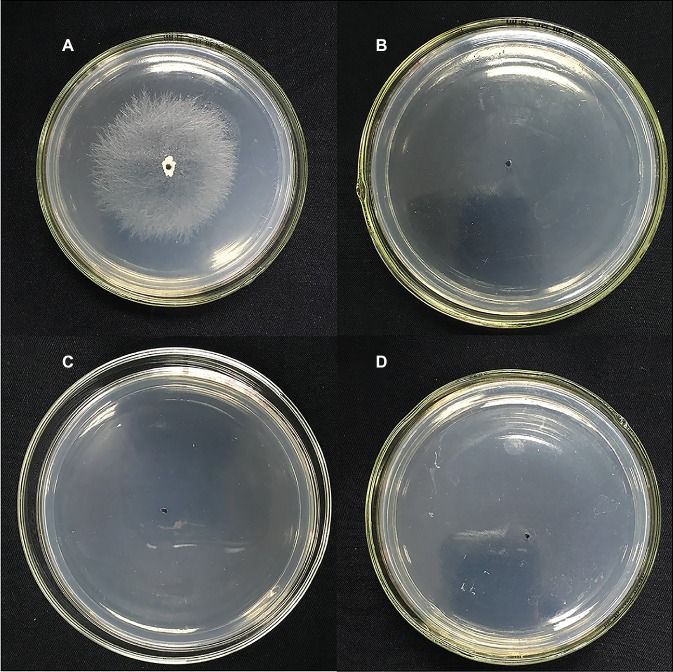
Effects of **NEP-1** on the sclerotial germination of *R. solani.* **(A)** Control plate; **(B)** plate treated with **NEP-1** at 15.00 μg/ml; **(C)** plate treated with **NEP-1** at 20.00 μg/ml; and **(D)** plate treated with **NEP-1** at 30.00 μg/ml.

Suppressing sclerotial formation and germination is a crucial strategy for control of *R. solani* (Soltani et al., 2017). Thus, we tested the capacity of **NEP-1** to suppress the sclerotial development of *R. solani*. As illustrated in Figure 5, on the plates with 40 μg/ml of **NEP-1**, few sclerotia were observed at the 6th day after inoculation and only a few abnormal sclerotium associated with large droplet secretion appeared after 12-day incubation. We further tested the effects of **NEP-1** on the sclerotial development of *R. solani* at low concentrations (15, 20, and 30 μg/ml) with results shown in Supplementary Figure S4. Compared with the control plates, sclerotial formation was considerably suppressed with an increase of the **NEP-1** concentration after 6 days of incubation. There were a very limited number of sclerotia occurring in the plates with 15.00 μg/ml of **NEP-1** (Supplementary Figure S4B). Meanwhile, the sclerotial maturation was also prevented by **NEP-1** and no mature sclerotia were observed when the concentration of **NEP-1** was above 20.00 μg/ml (Supplementary Figure S4). Moreover, **NEP-1** could also suppress the sclerotial germination significantly even at a very low concentration (15.00 μg/ml) (Figure 6). The above results revealed that **NEP-1** could inhibit the sclerotial development of *R. solani* including the formation, maturation, and germination, and that **NEP-1** might be an excellent antifungal agent for control of such sclerotia-forming fungal pathogens as *R. solani*.

### Effects of NEP-1 on Hyphal Morphology and Ultrastructure of *R. solani*

**Figure 7:**
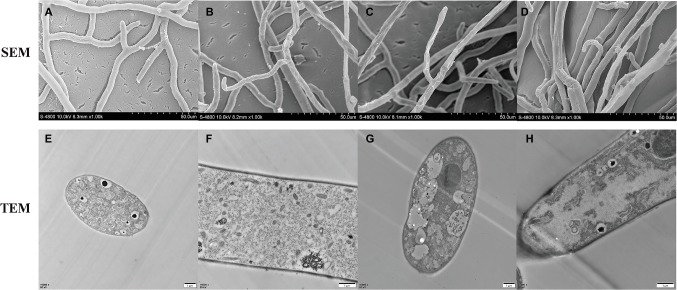
Effects of **NEP-1** on the hyphal morphology and ultrastructure of *R. solani*. **(A,E,F)** Healthy hyphae in control petri plates; **(B–D,G,H)** hyphae treated with **NEP-1** (40.00 μg/ml). Scale bar: 50.0 μm for SEM and 1 μm for TEM.

To better understand the mode of anti-fungal action of **NEP-1**, the effects of **NEP-1** on the hyphal morphology of *R. solani* were observed by SEM. As shown in Figure 7A, in the absence of **NEP-1**, the mycelia of *R. solani* exhibited a normal morphology of smooth, uniform, and robust hyphae with plump growing points. However, the **NEP-1** treated sample displayed an altered hyphal morphology characterized by irregular hyphae of the twisted, shriveled and distorted morphology, or with deformities at the growing points (Figures 7B–D).

TEM was employed to evaluate the ultrastructural alterations of *R. solani* with the results illustrated in Figure 7. The control sample represented a typical fungal ultrastructure of intact cell walls of normal thicknesses, evenly distributed cellular cytoplasm, and regularly shaped organelles in the mycelial cells (Figures 7E,F). For the sample treated with **NEP-1** (EC_50_ of 40.00 μg/ml), the mitochondrial abnormalities were clearly observed of hazy outlines and vacuolar degeneration, as well as the reduction in number (Figures 7G,H). Furthermore, the vacuolization and disorganization of the cytoplasm were also found in the mycelial cells exposed to **NEP-1** (Figures 7G,H).

## Discussion

*X. nematophila* YL001 is a valuable producer of natural compounds with pesticidal properties. Its fermentation broth was indicated to possess strong antimicrobial activity against some plant pathogens *in vitro* and *in vivo*, including *P. infestans* and *B. cinerea* (Guo et al., 2017; Zhang et al., 2018). Additionally, several pesticidal compounds have been identified from other *X. nematophila* stains, such as xenocoumacins (McInerney et al., 1991; Yang et al., 2011; Zhou et al., 2016), nematophin (Li et al., 1997a,b), PAX (peptide-antimicrobial-*Xenorhabdus*) peptides (Gualtieri et al., 2009; Fuchs et al., 2011), benzylideneacetone (Ji et al., 2004), xenortides, and xenematides (Lang et al., 2008). Xenocoumacins and nematophin were also present in the culture supernatant of *X. nematophila* YL001, which was confirmed by the HPLC-MS analysis in our previous study (Guo et al., 2017). Based on this, we try to isolate and identify new metabolites from the strain of *X. nematophila* YL001. In the present study, three dipeptide compounds were isolated and identified from the cell-free culture. **NEP-1** is an antibiotic which was first isolated from strain *X. nematophila* BC1 (Li et al., 1997a). **PDKP**, a cyclodipeptide of proline and glycine, has been isolated previously from a sea marine sponge of undescribed species of *Callyspongia* (Chen et al., 2014), as well as many microorganisms such as *Quambalaria cyanescens* (Bishay et al., 2018) and *Bacillus amyloliquefaciens* (Li et al., 2018). **MDKP**, a dimethylated cyclodipeptide of phenylalanine and leucine, was first synthesized by Nakao et al. (2016) and its core structure piperazine-2,5-dione was found in a variety of natural products from fungi, bacteria, plants, and mammals (Borthwick, 2012). Despite this, to the best of our knowledge, it was first identified as a natural compound in this study. Moreover, this is also the first report of the presence of **PDKP** and **MDKP** in the *X. nematophila* fermentation broth. To gain more information about their pesticidal properties, we also evaluated their antimicrobial activity against 10 common phytopathogens. **NEP-1** was demonstrated to possess a broad-spectrum antimicrobial activity *in vitro* (Table 1) and high control efficacy against *R. solani in vivo* (Figures 3, 4). Despite the weak antimicrobial activity against the selected plant pathogens, **PDKP** was determined to be a potent acaricide against *Tetranychus urticae* with a LC_50_ value of 95.96 μg/ml in a recent study (Li et al., 2018). **MDKP** was proved to have moderate antifungal activity against *E. turcicum* (Table 1). These results reveal the potential value of the three compounds in the crop protection.

**Figure 8:**
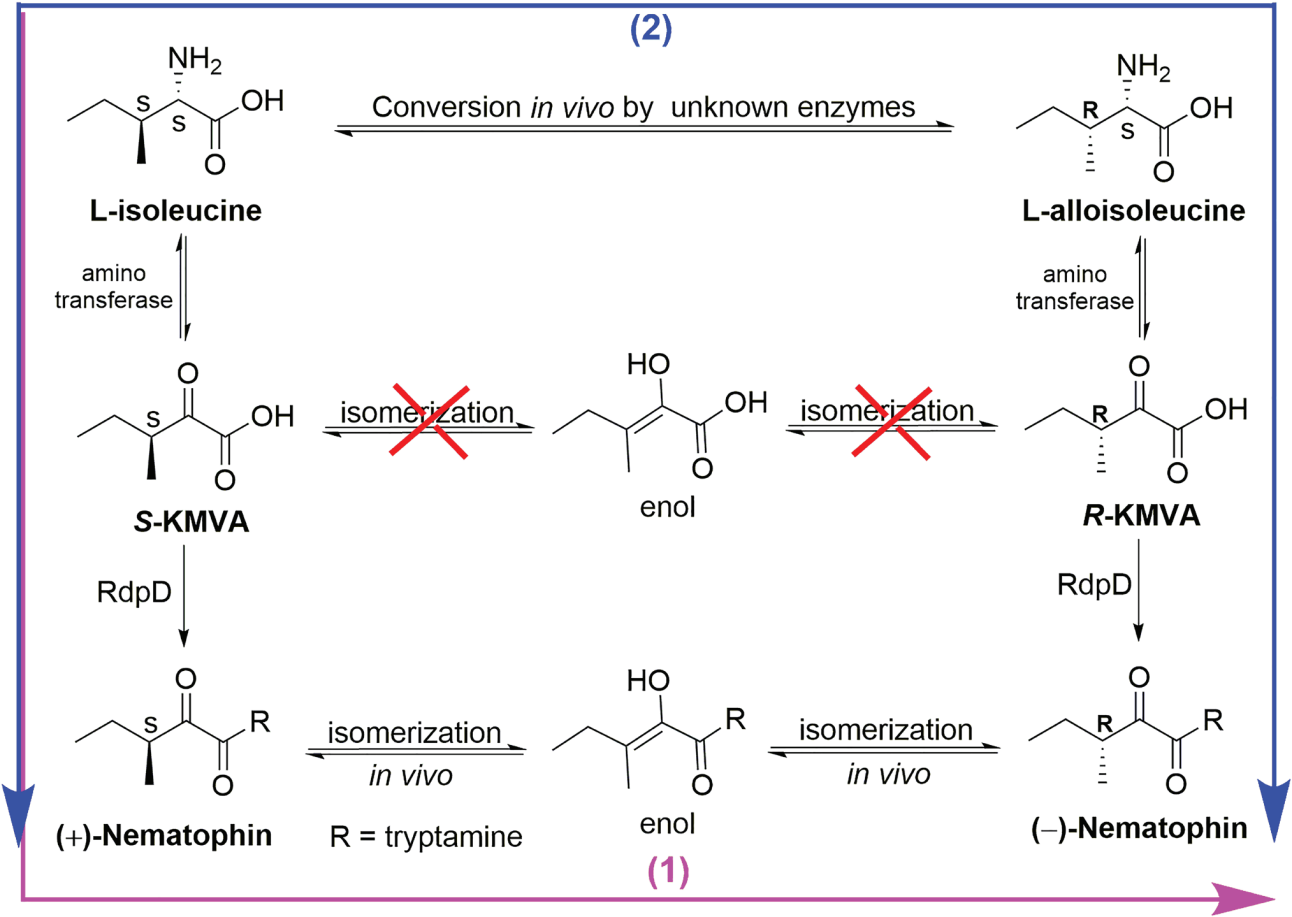
Two deductive pathways involved in the biosynthetic origin of (±)-nematophin (**NEP-1**).

The biosynthetic origin of (±)-nematophin (**NEP-1**) may be involved in two pathways in *X. nematophila* (Figure 8). The CD spectra (Supplementary Figure S2) and specific rotation of **NEP-1**, **-2**, and **-3** reveal that **NEP-1** is an enantiomeric mixture of nematophin, denying the previous inference that the natural nematophin is an enantiomer of (*S*)-configuration (Paik et al., 2003). Based on the biosynthetic process of nematophin, we deduced two possible pathways relevant to the formation of **NEP-1**. One is that (+)-nematophin (**NEP-3**) is derived from L-isoleucine (L-Ile) *via* the aminotransferase to generate 2-keto-3*S*-methylvaleric acid (*S*-KMVA) (Mamer, 2001), followed by enolization-induced racemization to form the nematophin stereoisomers *in vivo*. Previous studies revealed that the racemization of *S*-KMVA did not occur *in vivo*, and that L-alloisoleucine (L-allo-Ile) was derived from L-Ile rather than *R*-KMVA (Mamer and Reimer, 1992; Mamer and Lépine, 1996). Thus, the other pathway may be that **NEP-1** is derived from the authigenic L-Ile and L-allo-Ile, which can be converted to *S*-KMVA and *R*-KMVA, respectively. Conversion of L-Ile to L-allo-Ile was verified in bacteria, fungi, plants and mammalian systems and L-allo-Ile is also present in many natural cyclic peptide antibiotics (Mamer, 2001; Li et al., 2016), providing evidence for the second pathway. However, the actual mechanism of the racemization needs further clarification.

**NEP-1** has great potential to be developed as a new biopesticide for agricultural production. **NEP-1** not only represented broad-spectrum antimicrobial activity against 10 agricultural pathogenic fungi and oomycetes *in vitro* (Table 1) but also exerted a comparable control efficacy against *R. solani in vivo* at 1,000 μg/ml to **MBC** at 500 μg/ml on the rice plants (Figure 4). Moreover, **NEP-1** could also inhibit the sclerotial formation, maturation and germination of *R. solani* substantially even at a low concentration (15 μg/ml) (Supplementary Figures S4, S5), beneficial to control the disease extension during its epidemic period and its outbreak in the next year effectively. Besides high bioactivity (*in vitro* and *in vivo*), easy production at an industrial scale is also another key factor affecting the development of a microbial pesticide (Montesinos, 2003). **NEP-1** and its analogs can be synthesized through chemical methods due to its simple structure (Li et al., 1997b). In addition, researchers have also illuminated its biosynthesis pathway and achieved its heterologous production in *Escherichia coli* (Cai et al., 2017). In this paper, we also synthesized an enantiomeric mixture of nematophin (**NEP-2**) and its (*S*)-configuration structure (**NEP-3**), with high yields and easy operating conditions. The ultrastructure data suggest that **NEP-1** may diffuse into the exposed cells and then disrupt the cells by altering the subcellular structures, especially the mitochondrial structure, which are similar to those induced by camptothecin (**CPT**) against *R. solani*, indicating that they may possess a similar antifungal mechanism (Sirikantaramas et al., 2008; Dai et al., 2017). However, more work is needed to explore the effects of **NEP-1** on the mitochondria-related pathways of *R. solani* to further clarify its mechanism of action as a potent antifungal agent.

## Conclusions

Three dipeptide compounds were purified from the cultural broth and identified as (±)-nematophin (**NEP-1**), *cyclo* (L-Pro-Gly) and *N*,*N*′-dimethyl-*cyclo* (L-Phe-L-Leu). **NEP-1** demonstrated a wider antimicrobial spectrum than the other two compounds. It exhibited strong inhibitory effects on mycelial growth of *R. solani* and *P. infestans*. Its (*S*)-configuration structure (**NEP-3**, (+)-nematophin) was also synthesized and exhibited higher antifungal activity than the enantiomeric mixture. Besides, **NEP-1** not only possessed significant preventive and curative efficacy against *R. solani* on broad bean leaves, but also could effectively inhibit the disease extension on rice and broad bean plants. The sclerotial formation, maturation and germination of *R. solani* was also significantly inhibited by **NEP-1** at even a low concentration (15.00 μg/ml). The electron microscopic observations showed that the mycelium morphology of **R. solani** was adversely affected by nematophin at 40.00 μg/ml, as well as the subcellular structures. These results indicate that **NEP-1** has great potential to be used as a bio-pesticide in agricultural production.

## Data Availability

The raw data supporting the conclusions of this manuscript will be made available by the authors, without undue reservation, to any qualified researcher.

## Author Contributions

SZ, QL, YW, and XZ conceived and designed the experiments. SZ, QL, YH, JH, and ZY performed the experiments. QL and YH analyzed the data. JH and ZY contributed to reagents, materials, and analysis tools. SZ wrote the paper.

### Conflict of Interest Statement

The authors declare that the research was conducted in the absence of any commercial or financial relationships that could be construed as a potential conflict of interest.

## Footnotes

^1^https://imagej.nih.gov/ij/

## References

[ref1] Arfaoui A. El A. H. Daayf F. (2018). Pre-treatment of soybean plants with calcium stimulates ROS responses and mitigates infection by *Sclerotinia sclerotiorum*. Plant Physiol. Biochem. 122, 121–128. 10.1016/j.plaphy.2017.11.014, PMID: 29223021

[ref2] Bishay D. W. Abdel-Baky A. M. Moharram A. M. Malak L. G. Srivedavyasasri R. Ross S. A. (2018). Secondary metabolites from the fungus *Quambalaria cyanescens*. Chem. Nat. Compd. 54, 274–277. 10.1007/s10600-018-2322-2

[ref3] Borthwick A. D. (2012). 2,5-Diketopiperazines: synthesis, reactions, medicinal chemistry, and bioactive natural products. Chem. Rev. 112, 3641–3716. 10.1021/cr200398y, PMID: 22575049

[ref4] Böszörményi E. Ersek T. Fodor A. Fodor A. Foeldes L. S. Hevesi M.. (2009). Isolation and activity of *Xenorhabdus* antimicrobial compounds against the plant pathogens *Erwinia amylovora* and *Phytophthora nicotianae*. J. Appl. Microbiol. 107, 746–759. 10.1111/j.1365-2672.2009.04249.x, PMID: 19320949

[ref5] Cai X. Challinor V. L. Zhao L. Reimer D. Adihou H. Grün P.. (2017). Biosynthesis of the antibiotic nematophin and its elongated derivatives in entomopathogenic bacteria. Org. Lett. 19, 806–809. 10.1021/acs.orglett.6b03796, PMID: 28134534

[ref6] Campbell J. Lin Q. Geske G. D. Blackwell H. E. (2009). New and unexpected insights into the modulation of LuxR-type quorum sensing by cyclic dipeptides. ACS Chem. Biol. 4, 1051–1059. 10.1021/cb900165y, PMID: 19928886 PMC2801563

[ref7] Challinor V. L. Bode H. B. (2015). Bioactive natural products from novel microbial sources. Ann. N. Y. Acad. Sci. 1354, 82–97. 10.1111/nyas.12954, PMID: 26509922

[ref8] Chaston J. M. Suen G. Tucker S. L. Andersen A. W. Bhasin A. Bode E.. (2011). The entomopathogenic bacterial endosymbionts *Xenorhabdus* and *Photorhabdus*: convergent lifestyles from divergent genomes. PLoS One 6:e27909. 10.1371/journal.pone.0027909, PMID: 22125637 PMC3220699

[ref9] Chen Y. Peng Y. Gao C. Huang R. (2014). A new diketopiperazine from South China Sea marine sponge *Callyspongia* sp. Nat. Prod. Res. 28, 1010–1014. 10.1080/14786419.2014.903397, PMID: 24697743

[ref10] Crawford J. M. Portmann C. Zhang X. Roeffaers M. B. Clardy J. (2012). Small molecule perimeter defense in entomopathogenic bacteria. Proc. Natl. Acad. Sci. USA 109, 10821–10826. 10.1073/pnas.1201160109, PMID: 22711807 PMC3390839

[ref11] Dai T. T. Xu Z. Zhou X. Li B. Mao S. F. (2017). The inhibitory effect of the plant alkaloid camptothecin on the rice sheath blight pathogen *Rhizoctonia solani*. Int. J. Agric. Biol. 19, 558–562. 10.13227/j.hjkx.201704135, PMID: 29965427

[ref12] Dreyer J. Malan A. P. Dicks L. M. T. (2018). Bacteria of the genus *Xenorhabdus*, a novel source of bioactive compounds. Front. Microbiol. 9:3177. 10.3389/fmicb.2018.03177 30619229 PMC6305712

[ref13] Engel Y. Windhorst C. Lu X. Goodrichblair H. Bode H. B. (2017). The global regulators Lrp, LeuO, and HexA control secondary metabolism in entomopathogenic bacteria. Front. Microbiol. 8:209. 10.3389/fmicb.2017.00209 28261170 PMC5313471

[ref14] Fang X. Zhang M. Tang Q. Wang Y. Zhang X. (2014). Inhibitory effect of *Xenorhabdus nematophila* TB on plant pathogens *Phytophthora capsici* and *Botrytis cinerea in vitro* and *in planta*. Sci. Rep. 4, 1–7. 10.1038/srep04300, PMID: 24599183 PMC3944712

[ref15] Feng S. Shu C. Wang C. Jiang S. Zhou E. (2016). Survival of *Rhizoctonia solani* AG-1 IA, the causal agent of rice sheath blight, under different environmental conditions. J. Phytopathol. 165, 44–52. 10.1111/jph.12535

[ref16] Fuchs S. W. Proschak A. Jaskolla T. W. Karas M. Bode H. B. (2011). Structure elucidation and biosynthesis of lysine-rich cyclic peptides in *Xenorhabdus nematophila*. Org. Biomol. Chem. 9, 3130–3132. 10.1039/c1ob05097d, PMID: 21423922

[ref17] Gualtieri M. Aumelas A. Thaler J.-O. (2009). Identification of a new antimicrobial lysine-rich cyclolipopeptide family from *Xenorhabdus nematophila*. J. Antibiot. 62, 295–302. 10.1038/ja.2009.31, PMID: 19373275

[ref18] Guo S. Zhang S. Fang X. Liu Q. Gao J. Bilal M. (2017). Regulation of antimicrobial activity and xenocoumacins biosynthesis by pH in *Xenorhabdus nematophila*. Microb. Cell Factories 16:203. 10.1186/s12934-017-0813-7 PMC5688692 29141647

[ref19] Hazir S. Shapiro-Ilan D. I. Bock C. H. Hazir C. Leite L. G. Hotchkiss M. W. (2016). Relative potency of culture supernatants of *Xenorhabdus* and *Photorhabdus* spp. on growth of some fungal phytopathogens. Eur. J. Plant Pathol. 146, 1–13. 10.1007/s10658-016-0923-9

[ref20] Helge B. (2011). Structure elucidation and biosynthesis of lysine-rich cyclic peptides in *Xenorhabdus nematophila*. Org. Biomol. Chem. 9, 3130–3132. 10.1039/c1ob05097d, PMID: 21423922

[ref21] Houard J. Aumelas A. Noël T. Pages S. Gualtieri M. (2013). Cabanillasin, a new antifungal metabolite, produced by entomopathogenic *Xenorhabdus cabanillasii* JM26. J. Antibiot. 66, 617–620. 10.1038/ja.2013.58, PMID: 23756685

[ref22] Ji D. Yi Y. Kang G. H. Choi Y. H. Kim P. Baek N. I.. (2004). Identification of an antibacterial compound, benzylideneacetone, from *Xenorhabdus nematophila* against major plant-pathogenic bacteria. FEMS Microbiol. Lett. 239, 241–248. 10.1016/j.femsle.2004.08.041, PMID: 15476972

[ref23] Kanagaraj Muthu-Pandian C. Sengottayan S.-N. Vel T. Karthi S. Sreenath Kumar C. Sreenath Kumar C. (2018). Bacterial compounds, as biocontrol agent against early blight (*Alternaria solani*) and tobacco cut worm (*Spodoptera litura* Fab.) of tomato (*Lycopersicon esculentum* Mill.) Arch. Phytopathol. Plant Protect. 51, 729–753. 10.1080/03235408.2018.1496525

[ref24] Kazempour M. N. (2004). Biological control of *Rhizoctonia solani*, the causal agent of rice sheath blight by antagonistics bacteria in greenhouse and field conditions. Plant Pathol. J. 3, 88–96. 10.3923/ppj.2004.88.96

[ref25] Kim B. S. Hwang B. K. (2007). Microbial fungicides in the control of plant diseases. J. Phytopathol. 155, 641–653. 10.1111/j.1439-0434.2007.01314.x

[ref26] Kumar V. V. (2018). “Biofertilizers and biopesticides in sustainable agriculture” in Role of rhizospheric microbes in soil: Volume 1: Stress management and agricultural sustainability. ed. Meena V. S. (Singapore: Springer Singapore), 377–398.

[ref27] Lang G. Kalvelage T. Peters A. Wiese J. Imhoff J. F. (2008). Linear and cyclic peptides from the entomopathogenic bacterium *Xenorhabdus nematophilus*. J. Nat. Prod. 71, 1074–1077. 10.1021/np800053n, PMID: 18491867

[ref28] Lengai G. M. W. Muthomi J. W. (2018). Biopesticides and their role in sustainable agricultural production. J. Biosci. Med. 6, 7–41. 10.4236/jbm.2018.66002

[ref29] Lewis J. A. Lumsden R. D. (2001). Biocontrol of damping-off of greenhouse-grown crops caused by *Rhizoctonia solani* with a formulation of *Trichoderma* spp. Crop Prot. 20, 49–56. 10.1016/S0261-2194(00)00052-1

[ref30] Li J. Chen G. Webster J. M. (1997a). Nematophin, a novel antimicrobial substance produced by *Xenorhabdus nematophilus* (Enterobactereaceae). Can. J. Microbiol. 43, 770–773. 10.1139/m97-110 9304787

[ref31] Li J. Chen G. Webster J. M. (1997b). Synthesis and antistaphylococcal activity of nematophin and its analogues. Bioorg. Med. Chem. Lett. 7, 1349–1352. 10.1016/s0960-894x(97)00223-0

[ref32] Li Q. Qin X. Liu J. Gui C. Wang B. Li J.. (2016). Deciphering the biosynthetic origin of l-allo-isoleucine. J. Am. Chem. Soc. 138, 408–415. 10.1021/jacs.5b11380, PMID: 26669414

[ref33] Li X. Y. Wang Y. H. Yang J. Cui W. Y. He Y. Q. (2018). Acaricidal activity of cyclodipeptides from *Bacillus amyloliquefaciens* W1 against *Tetranychus urticae*. J. Agric. Food Chem. 66, 10163–10168. 10.1021/acs.jafc.8b03806, PMID: 30200767

[ref34] Mamer O. A. (2001). Initial catabolic steps of isoleucine, the R-pathway and the origin of alloisoleucine. J. Chromatogr. B Biomed. Sci. Appl. 758, 49–55. 10.1016/S0378-4347(01)00111-6, PMID: 11482734

[ref35] Mamer O. A. Lépine F. L. (1996). ^15^N conservation in the metabolic conversion of isoleucine to alloisoleucine in the rat. J. Mass Spectrom. 31, 1382–1388. 10.1002/(SICI)1096-9888(199612)31:12<1382::AID-JMS435>3.0.CO;2-V, PMID: 8990521

[ref36] Mamer O. A. Reimer M. L. (1992). On the mechanisms of the formation of L-alloisoleucine and the 2-hydroxy-3-methylvaleric acid stereoisomers from L-isoleucine in maple syrup urine disease patients and in normal humans. J. Biol. Chem. 267, 22141–22147. 10.1016/S0022-5193(05)80771-4, PMID: 1429566

[ref37] Mayo S. Gutiérrez S. Malmierca M. G. Lorenzana A. Campelo M. P. Hermosa R.. (2015). Influence of *Rhizoctonia solani* and *Trichoderma* spp. in growth of bean (*Phaseolus vulgaris* L.) and in the induction of plant defense-related genes. Front. Plant Sci. 6:685. 10.3389/fpls.2015.00685, PMID: 26442006 PMC4584982

[ref38] McInerney B. V. Taylor W. C. Lacey M. J. Akhurst R. J. Gregson R. P. (1991). Biologically active metabolites from *Xenorhabdus* spp., part 2. Benzopyran-1-one derivatives with gastroprotective activity. J. Nat. Prod. 54, 785–795. 10.1021/np50075a006, PMID: 1955881

[ref39] Mnif I. Ghribi D. (2015). Potential of bacterial derived biopesticides in pest management. Crop Prot. 77, 52–64. 10.1016/j.cropro.2015.07.017

[ref40] Moni Z. R. Ali M. A. Alam M. S. Rahman M. A. Bhuiyan M. R. Mian M. S. (2016). Morphological and genetical variability among *Rhizoctonia solani* isolates causing sheath blight disease of rice. Rice Sci. 23, 42–50. 10.1016/j.rsci.2016.01.005

[ref41] Montesinos E. (2003). Development, registration and commercialization of microbial pesticides for plant protection. Int. Microbiol. 6, 245–252. 10.1007/s10123-003-0144-x, PMID: 12955583

[ref42] Nakao M. Hiroyama Y. Fukayama S. Sano S. (2016). N4-methylation changes the conformation of (3S,6S)-3-alkyl-6-benzylpiperazine-2,5-diones from folded to extended. J. Mol. Struct. 1116, 37–44. 10.1016/j.molstruc.2016.03.019

[ref43] Nicolopoulou-Stamati P. Maipas S. Kotampasi C. Stamatis P. Hens L. (2016). Chemical pesticides and human health: the urgent need for a new concept in agriculture. Front. Public Health 4:148. 10.3389/fpubh.2016.00148 27486573 PMC4947579

[ref44] Paik S. Park M. K. Jhun S. H. Park H. K. Suh S. I. (2003). Isolation and synthesis of tryptamine derivatives from a symbiotic bacterium *Xenorhabdus nematophilus* PC. ChemInform 34, 2101–2118. 10.1002/chin.200345209

[ref45] Pantel L. Florin T. Dobosz-Bartoszek M. Racine E. Sarciaux M. Serri M.. (2018). Odilorhabdins, antibacterial agents that cause miscoding by binding at a new ribosomal site. Mol. Cell 70:83. 10.1016/j.molcel.2018.03.001, PMID: 29625040

[ref46] Park D. S. Sayler R. Hong Y. G. Nam M. H. Yang Y. (2008). A method for inoculation and evaluation of rice sheath blight disease. Plant Dis. 92, 25–29. 10.1094/PDIS-92-1-0025, PMID: 30786366

[ref47] Rohr J. R. Brown J. Battaglin W. A. McMahon T. A. Relyea R. A. (2017). A pesticide paradox: fungicides indirectly increase fungal infections. Ecol. Appl. 27, 2290–2302. 10.1002/eap.1607, PMID: 28763165 PMC5711531

[ref48] Sharma K. Walia S. Ganguli S. Kundu A. (2016). Analytical characterization of secondary metabolites from Indian *Xenorhabdus* species the symbiotic bacteria of entomopatathogenic nematode (*Steinernema* spp.) as antifungal agent. Natl. Acad. Sci. Lett. 39, 175–180. 10.1007/s40009-016-0453-1

[ref49] Sirikantaramas S. Yamazaki M. Saito K. (2008). Mutations in topoisomerase I as a self-resistance mechanism coevolved with the production of the anticancer alkaloid camptothecin in plants. Proc. Natl. Acad. Sci. USA 105, 6782–6786. 10.1073/pnas.0801038105, PMID: 18443285 PMC2365566

[ref50] Soltani N. M. Ghs B. Khatami M. Amini A. Aghighi S. (2017). *In vitro* and *in vivo* antifungal properties of silver nanoparticles against *Rhizoctonia solani*, a common agent of rice sheath blight disease. IET Nanobiotechnol. 11, 236–240. 10.1049/iet-nbt.2015.0121, PMID: 28476979 PMC8676185

[ref51] Soylu E. M. Kurt S. Soylu S. (2010). *In vitro* and *in vivo* antifungal activities of the essential oils of various plants against tomato grey mould disease agent *Botrytis cinerea*. Int. J. Food Microbiol. 143, 183–189. 10.1016/j.ijfoodmicro.2010.08.015, PMID: 20826038

[ref52] Townsend B. B. Willetts H. J. (1954). The development of sclerotia of certain fungi. Trans. Br. Mycol. Soc. 37, 213–221. 10.1016/S0007-1536(54)80003-9

[ref53] Wu Z. M. Yang Y. Li K. T. (2019). Antagonistic activity of a novel antifungalmycin N2 from *Streptomyces* sp. N2 and its biocontrol efficacy against *Rhizoctonia solani*. FEMS Microbiol. Lett. 366, 1–8. 10.1093/femsle/fnz018, PMID: 30689866

[ref54] Yang X. Qiu D. Yang H. Liu Z. Zeng H. Yuan J. (2011). Antifungal activity of xenocoumacin 1 from *Xenorhabdus nematophilus* var. pekingensis against *Phytophthora infestans*. World J. Microbiol. Biotechnol. 27, 523–528. 10.1007/s11274-010-0485-5

[ref55] Zhang S. Fang X. Tang Q. Ge J. Wang Y. Zhang X. (2018). CpxR negatively regulates the production of xenocoumacin 1, a dihydroisocoumarin derivative produced by *Xenorhabdus nematophila*. Microbiology 8:e00674. 10.1002/mbo3.674 PMC6391269 29888873

[ref56] Zheng A. Lin R. Zhang D. Qin P. Xu L. Peng A. (2013). The evolution and pathogenic mechanisms of the rice sheath blight pathogen. Nat. Commun. 4:1424. 10.1038/ncomms2427 23361014 PMC3562461

[ref57] Zhou S. Liu Y. Zhang M. Li B. Chen X. Liang W. (2016). Comparison of the virulence and cognate virulence factors of multinucleate, binucleate and uninucleate *Rhizoctonia* isolates, causing sheath blight on maize plants. Eur. J. Plant Pathol. 145, 501–506. 10.1007/s10658-015-0855-9.aa

[ref58] Zhou T. Yang X. Qiu D. Zeng H. (2016). Inhibitory effects of xenocoumacin 1 on the different stages of *Phytophthora capsici* and its control effect on *Phytophthora* blight of pepper. BioControl 62, 151–160. 10.1007/s10526-016-9779-3

