# Supplementary material for: Nematophin, an Antimicrobial Dipeptide Compound From Xenorhabdus nematophila YL001 as a Potent Biopesticide for Rhizoctonia solani Control
Source: Front Microbiol. 2019 Aug 7;10:1765. doi: 10.3389/fmicb.2019.01765 (PMC6693444; doi:10.3389/fmicb.2019.01765)
Supplement: Supplementary file 1 [file Data_Sheet_1.pdf]

Supporting Information

Nematophin, an antimicrobial dipeptide compound from *Xenorhabdus nematophila* YL001 as a potent biopesticide for *Rhizoctonia solani* control

Shujing Zhang[1], *Qi Liu*[2], *Yunfei Han*[1], *Jinghua Han*[1], *Yonghong Wang*[1]* *and Xing Zhang*[1]

[1]Research and Development Center of Biorational Pesticides, Key Laboratory of Plant Protection Resources and Pest Management of Ministry of Education, College of Plant Protection, Northwest A&F University, 22 Xinong Road, Yangling, Shaanxi 712100, China

[2]Plant quarantine and protection bureau of Zhumadian, Henan 463000, China

[*]Correspondence:

Yonghong Wang (yhwang@nwafu.edu.cn)

Table of contents

1. **Synthesis of (±)-Nematophin and (+)-Nematophin**

2. **Biochemical and molecular characteristics of *X. nematophila* YL001 (Table S1 and S2; Figure S1)**

3. **Table S3**

4. **Figure S2 and Figure S3**

5. **^{1}H NMR, ^{13}C NMR and MS spectra (Figure S4-S28)**

1. Synthesis of (±)-Nematophin

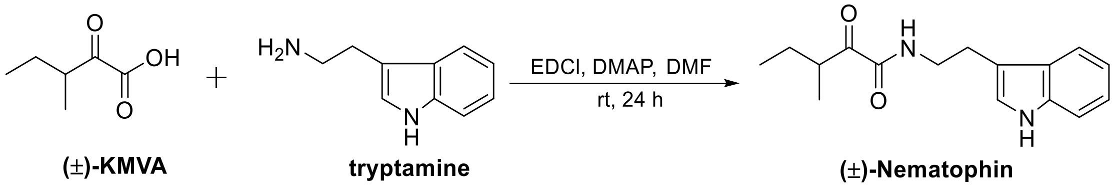

Scheme S1. Synthetic route for the preparation of the target compound (±)-Nematophin

Ethyl-dimethylaminopropyl-carbodiimide hydrochloride (EDCl, 2.4 mmol, 1.2 equiv) and N, N-dimethyl-4-aminopyridine (DMAP, 0.4 mmol, 0.2 equiv) were add into an ice-cooled solution of (±)-2-keto-3-methylvaleric acid ((±)-KMVA, 2 mmol, 260.3 mg) in N, N-dimethyformamide (DMF, 5 mL) and the mixture was stirred at 0 ℃ for 30 min. Then, tryptamine (2.4 mmol, 1.2 equiv) was added into the mixture. After being stirred at 0 ℃ for another 30 min, the mixture was allowed to warm to 25 ℃ and then stirred for 24 h. The resulting solution was added into 20 mL of distilled water and extracted with ethyl acetate (3 × 20 mL). The organic layer was collected, dried over anhydrous Na_2SO_4, filtered, and concentrated *in vacuo* to afford a yellow residue. The residue was purified by silica gel chromatography (petroleum ether/EtOAc, 5:1, V/V) to yield (±)-Nematophin (0.34 g, 62.5%) as a white solid. ^{1}H NMR (500 MHz, $CDCl_3$) δ 8.15 (bs, 1H), 7.65 (d, *J* = 7.9 Hz, 1H), 7.42 (d, *J* = 8.1 Hz, 1H), 7.26 (t, *J* = 7.6 Hz, 1H), 7.18 (t, *J* = 7.5 Hz, 1H), 7.11 (bs, 1H), 7.08 (d, *J* = 1.5 Hz, 1H), 3.69 (q, *J* = 6.7 Hz, 2H), 3.59 – 3.49 (m, 1H), 3.07 (t, *J* = 6.9 Hz, 2H), 1.83 – 1.71 (m, 1H), 1.50 – 1.37 (m, 1H), 1.13 (d, *J* = 6.9 Hz, 3H), 0.94 (t, *J* = 7.4 Hz, 3H); ^{13}C NMR (125 MHz, $CDCl_3$) δ 202.39, 160.08, 136.47, 127.18, 122.34, 122.04, 119.61, 118.67, 112.56, 111.30, 40.40, 39.55, 25.46, 25.19, 15.17, 11.50; ESI-MS (m/z): $[M+Na]^+$ calculated for

$C_{16}H_{20}N_2NaO_2$ 295.14, found 295.10; $[M–H]^-$ calculated for $C_{16}H_{19}N_2O_2$ 271.14, found 271.11.

2. Synthesis of (+)-Nematophin

2.1 (2*S*, 3*S*)-2-Hydroxy-3-methylpentanoic Acid (a) (Paik et al., 2003)

6.2 g of $NaNO_2$ (89.8 mmol) was added into an ice-cooled (0 ℃) solution of L-isoleucine (1.96 g, 15 mmol) in 0.5 M H_2SO_4 (60 mL) over a period of 1 h. After that, the mixture was allowed to warm to 25 ℃ and stirred for another 24 h. The resulting solution was extracted with ether (3 × 20 mL). Then, the organic extracts were collected, dried by anhydrous Na_2SO_4, filtered, and evaporated *in vacuo* to give a yellow oil. Purification was performed by crystallization from ether and petroleum ether to give **a** (1.5 g, 75.8%) as a white solid. $[\alpha]_D^{25}$: 20.18 (c 4, $CHCl_3$); ^1H NMR (500 MHz, $CDCl_3$) δ 4.19 (d, *J* = 3.4 Hz, 1H), 1.84 – 1.96 (m, 1H), 1.50 – 1.38 (m, 1H), 1.37 – 1.22 (m, 1H), 1.03 (d, *J* = 6.9 Hz, 3H), 0.93 (t, *J* = 7.4 Hz, 3H); ^{13}C NMR (125 MHz, $CDCl_3$) δ 179.58, 74.66, 38.91, 23.68, 15.34, 11.73.

2.2 (2'*S*, 3'*S*)-N-(Indol-3-ylethyl)-2'-hydroxy-3'-methylpentan-amide (b)

The synthetic procedure of **b** was identical to that of (±)-Nematophin except N-Hydroxysuccinimide (NHS, 1.2 equiv) and 2-hydroxy-isoleucic acid (**a**, 1 equiv) being used. The crude product was purified by chromatography on silica gel (CH_2Cl_2/MeOH, 50:1, V/V) to yield **b** (0.28 g, 61.3%) as a white solid. $[\alpha]_D^{25}$: −4.51 (c 1.55, $CHCl_3$); ^1H NMR (500 MHz, $CDCl_3$) δ 8.19 (bs, 1H), 7.60 (d, *J* = 7.8 Hz, 1H), 7.35 (d, *J* = 8.1 Hz, 1H), 7.20 (t, *J* = 7.3 Hz, 1H), 7.12 (t, *J* = 7.3 Hz, 1H), 6.99 (d, *J* = 2.2 Hz 1H), 6.54 (bs,

1H), 3.90 (dd, *J* = 5.0, 3.5 Hz, 1H), 3.62 (q, *J* = 6.6 Hz, 2H), 3.03 – 2.91 (m, 2H), 2.73 (d, *J* = 5.3 Hz, 1H), 1.86 – 1.76 (m, 1H), 1.39 – 1.27 (m, 1H), 1.17 – 1.04 (m, 1H), 0.94 (d, *J* = 6.9 Hz, 3H), 0.85 (t, *J* = 7.4 Hz, 3H); ^{13}C NMR (125 MHz, $CDCl_3$) δ 173.27, 136.44, 127.32, 122.22, 122.09, 119.47, 118.68, 112.83, 111.34, 76.36, 39.31, 38.77, 25.44, 23.06, 15.61, 11.88; ESI-MS (m/z): $[M+Na]^+$ calculated for $C_{16}H_{22}N_2NaO_2$ 297.15, found 297.10.

2.3 (+)-Nematophin (Paik et al., 2003)

200 mg of Dess-Martin reagent (periodinane, 0.47 mmol) was added into a solution of **b** (100 mg, 0.36 mmol) in 5 mL of CH_2Cl_2. The mixture was stirred for 20 min at 25 ℃. The resulting solution was filtered through Celite, and the solvent was removed by vacuum rotary evaporation. The residue was purified on a silica gel column eluted with a petroleum ether-EtOAc mixture (5:1, V/V) to afford 49.2 mg (50.3%) of (+)-Nematophin as a white solid. $[\alpha]_D^{25}$: 31.66 (c 0.58, $CHCl_3$); ^1H NMR (500 MHz, $CDCl_3$) δ 8.11 (bs, 1H), 7.60 (d, *J* = 7.9 Hz, 1H), 7.37 (d, *J* = 8.1 Hz, 1H), 7.21 (t, *J* = 7.3 Hz, 1H), 7.13 (t, *J* = 7.4 Hz, 1H), 7.05 (bs, 1H), 7.03 (d, *J* = 1.7 Hz, 1H), 3.64 (q, *J* = 6.7 Hz, 2H), 3.50 (h, *J* = 6.8 Hz, 1H), 3.02 (t, *J* = 6.9 Hz, 2H), 1.78 – 1.66 (m, 1H), 1.45 – 1.33 (m, 1H), 1.08 (d, *J* = 6.9 Hz, 3H), 0.88 (t, *J* = 7.4 Hz, 3H); ^{13}C NMR (125 MHz, $CDCl_3$) δ 202.40, 160.07, 136.46, 127.17, 122.34, 122.07, 119.61, 118.68, 112.53, 111.32, 40.40, 39.55, 25.47, 25.20, 15.19, 11.54; ESI-MS (m/z): $[M+H]^+$ calculated for $C_{16}H_{21}N_2O_2$ 273.16, found 273.00; $[M+Na]^+$ calculated for $C_{16}H_{20}N_2NaO_2$ 295.14, found 295.07; $[M–H]^–$ calculated for $C_{16}H_{19}N_2O_2$ 271.14, found 271.10.

Table S1 Morphological characteristics of *X. nematophila* YL001 (Wang and Zhang, 2006)

Symbiotic bacteria	Gram	shape	Size (μm)	Spore
X. nematophilus YL001	G^-	Rod	0. 5×1. 3-1. 7×9. 5	None

Table S2 Biochemical characteristics of *X. nematophila* YL001 (Wang and Zhang, 2006)

Physiological index	Positive / negative reaction
Catalase	-
Oxidase	-
Urease	-
Lecithinase	+
Protease	+
Gelatin	+
Lipase	-
Tween 80	-
Nitrate reduction	-
Indole production	-
H_2S production	-
Voges-Prokauer test	-
Starch hydrolysis-soluble	-
Phenylalanine deaminase	-
Tryptophan deaminase	-
Aesculin hydrolysis	-
D, L -Glycerate	+
Simmon’s citrate	+

Note: +: positive reaction ; -. negative reaction;

Xenorhabdus nematophila strain YL001 16S ribosomal RNA gene, partial sequence

AGATTGAACGCTGGCGGCAGGCCTAACACATGCAAGTCGGACGGTAACAGGAAA
CAGCTTGCTGTTTTGCTGACGAGTGGCGGACGGGTGAGTAATGTCTGGGGATCTGCCC
GATGGAGGGGGATAACCACTGGAAACGGTGGCTAATACCGCATGACCTCTTGGGAGTA
AAGTGGGGGACCTTCGGGCCTCACGCCATCGGATGAACCCAGATGGGATTAGCTAGTA
GGCGGGGTAATGGCCCACCTAAGCGACGATCCCTAGCTGGTCTGAGAGGATGACCAGC
CACACTGGGACTGAGACACGGCCCAGACTCCTACGGGAGGCAGCAGTGGGGAATATT
GCACAATGGGCGCAAGCCTGATGCAGCCATGCCGCGTGTATGAAGAAGGCCTTCGGGT
TGTAAAGTACTTTCAGCGGGGAGGAAGGCGTAAGTCTGAACAGGGCTTACGATTGACG
TTACCCGCAGAAGAAGCACCGGCTAACTCCGTGCCAGCAGCCGCGGTAATACGGAGG
GTGCAAGCGTTAATCGGAATTACTGGGCGTAAAGCGCACGCAGGCGGTCAATTAAGTT
GGATGTGAAATCCCCGGGCTTAACCCAGGAACGGCATCCAAGACTGGTTGGCTAGAGT
CTCGTAGAGGGGGGTAGAATTCCACGTGTAGCGGTGAAATGCGTAGAGATGTGGAGGA
ATACCGGTGGCGAAGGCGGCCCCCTGGACGAAGACTGACGCTCAGGTGCGAAAGCGT
GGGGAGCAAACAGGATTAGATACCCTGGTAGTCCACGCTGTAAACGATGTCGATTTGG
AGGCTGTGCCCTTGAGGCGTGGCTTCCGGAGCTAGCGCGTTAAATCGACCGCCTGGGG
AGTACGGCCGCAAGGTTAAAACTCAAATGAATTGACGGGGGCCCGCACAAGCGGTGG
AGCATGTGGTTTAATTCGATGCAACGCGAAGAACCTTACCTACTCTTGACATCCACGGG
ATCAGGCAGAGATGCCGGAGTGCCTTCGGGAACCGTGAGACAGGTGCTGCATGGCTG
TCGTCAGCTCGTGTTGTGAAATGTTGGGTTAAGTCCCGCAACGAGCGCAACCCTTATC
CTTTGTTGCCAGCACTTCGGGTGGGAACTCAAGGGAGACTGCCGGTGATAAACCGGA
GGAAGGTGGGGATGACGTCAAGTCATCATGGCCCTTACGAGTAGGGCTACACACGTGC
TACAATGGCAGATACAAAGAGAAGCGACCTCGCGAGAGCAAGCGGAACTCATAAAGT
CTGTCGTAGTCCGGATTGGAGTCTGCAACTCGACTCCATGAAGTCGGAATCGCTAGTA
ATCGTAGATCAGAATGCTACGGTGAATACGTTCCCGGGCCTTGTACACACCGCCCGTCA
CACCATGGGAGTGGGTTGCAAAAGAAGTAGGTAGCTTAACCTTCGGGGGGGCGCTTAC
CACTTTGTGATTCATGACTGGGGTGAAGTCGTAACAAGGTAACCGTAGGGGAACC

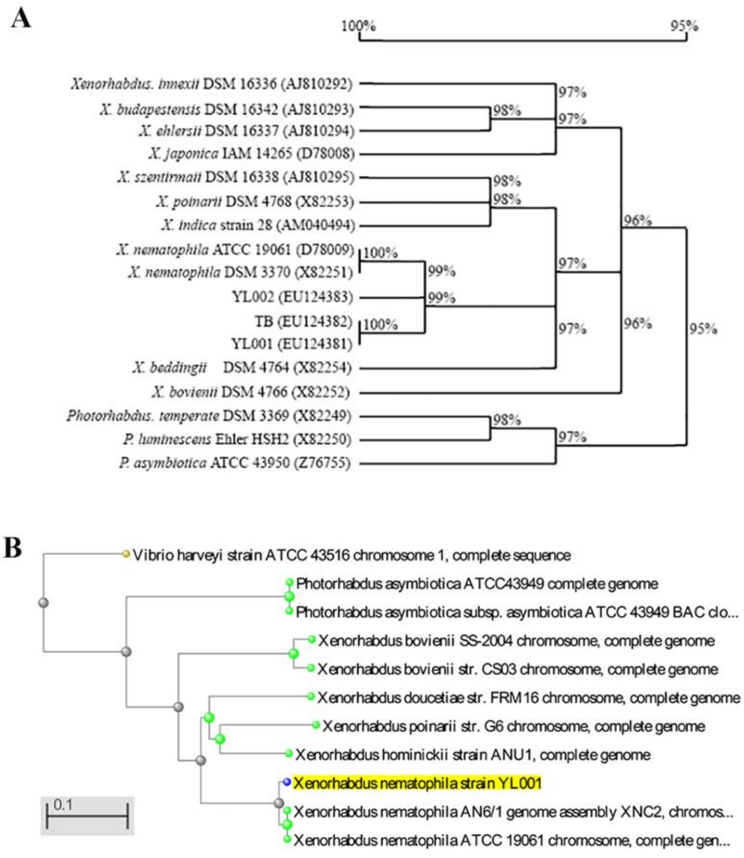

Figure S1 The homology trees of *Xenorhabdus nematophila* YL001 based on 16S rDNA (A) and the genome sequence (B).

Table S3 Pathogenic fungi and oomycetes used in this study

Pathogenic fungi	Host plant	Geographic origins
Rhizoctonia solani	Rice	Hanzhong (Shannxi province)
Exserohilum turcicum	Corn	Yangling (Shannxi province)
Phytophthora infestans	Potato	Yulin (Shannxi province)
Fusarium graminearum	Wheat	Yangling (Shannxi province)
Verticillium dahliae	cucumber	Yangling (Shannxi province)
Phytophthora capsici	Pepper	Yangling (Shannxi province)
Botrytis cinerea	Tomato	Yangling (Shannxi province)
Sclerotinia sclerotiorum	Oilseed rape	Hanzhong (Shannxi province)
Alternaria alternate	tobacco	Mianchi (Henan province)
Gaeumannomyces gramini	Wheat	Yangling (Shannxi province)

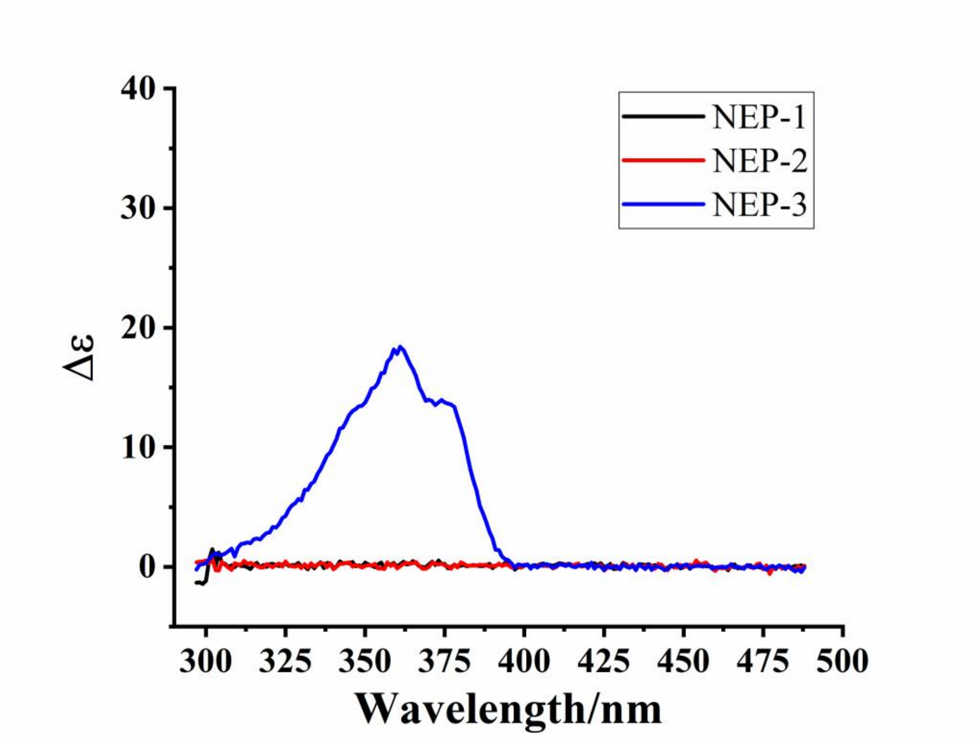

Figure S2 The circular dichroism (CD) spectra of **NEP-1**, **-2** and **-3** in $CHCl_3$ with a concentration of 5.8 mg/mL at 25 ℃.

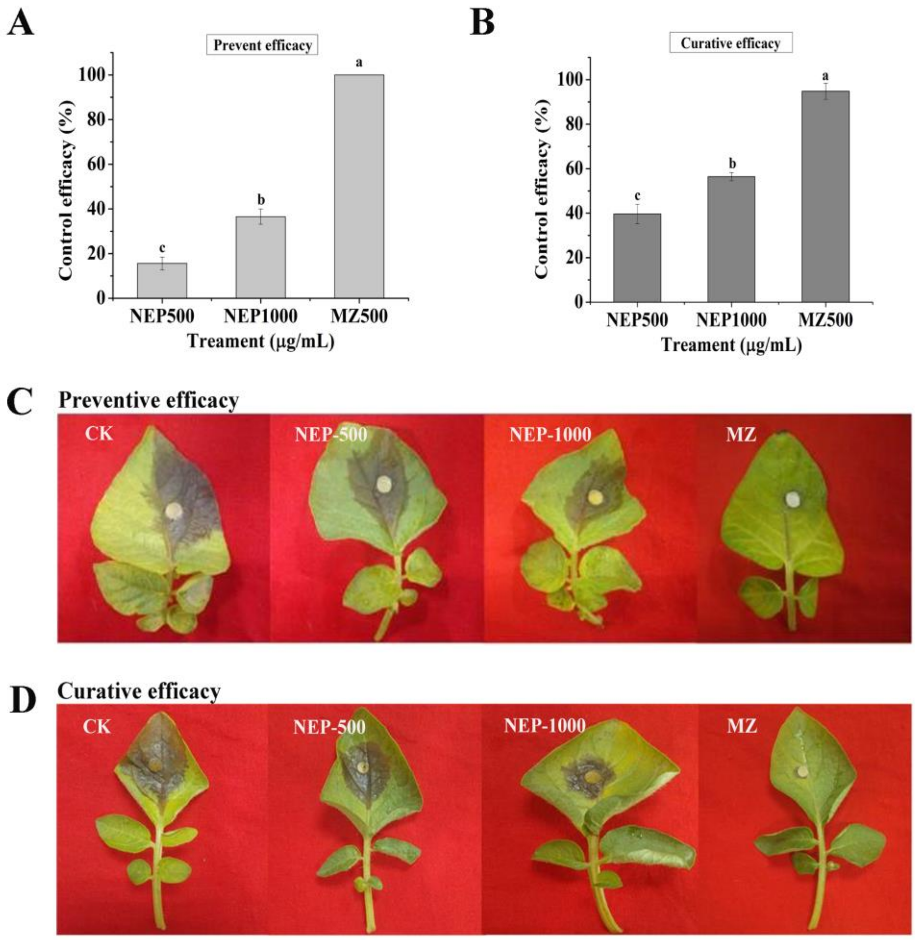

Figure S3 *In vivo* antimicrobial activity of **NEP-1** and mancozeb against *P. infestans*. **(A, C)** Protective activity of **NEP-1**; **(B, D)** Curative activity of **NEP-1**. Preventive/curative efficacy: the control efficacy of a compound that was sprayed on potato leaves 12 h before/after inoculation with *P. infestans*. NEP500, 500 μg/mL of **NEP-1**; NEP1000, 1000 μg/mL of **NEP-1**; MZ: 500 μg/mL of mancozeb.

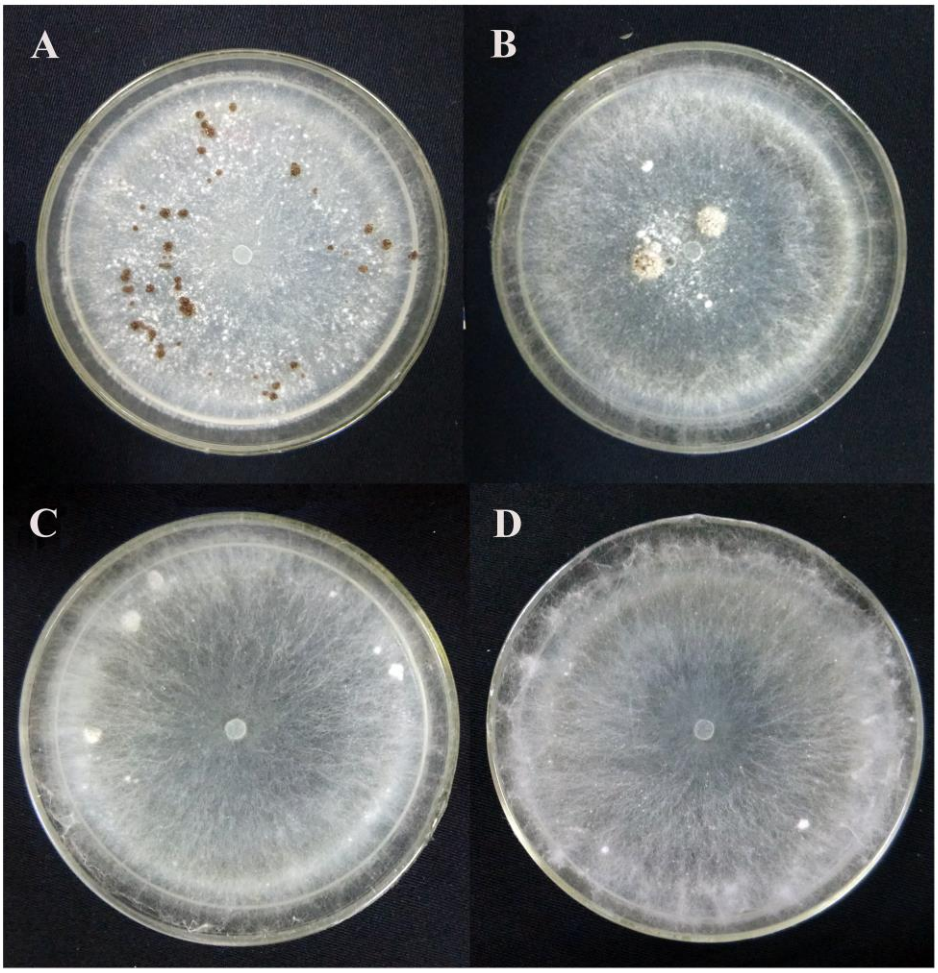

Figure S4 Effect of **NEP-1** on sclerotial formation of *R. solani*. (A) Control plate; (B) Plate treated with **NEP-1** at 15.00 μg/mL; (C) Plate treated with **NEP-1** at 20.00 μg/mL; (D) Plate treated with **NEP-1** at 30.00 μg/mL. All the plates were incubated under identical conditions for 6 days.

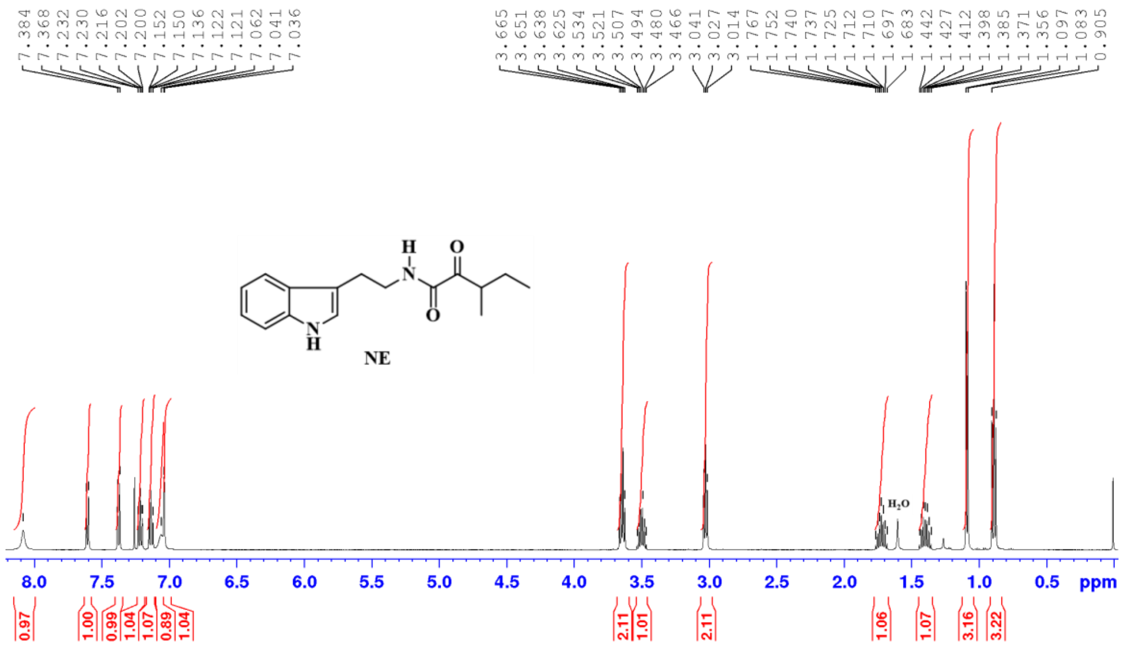

Figure S5 ^{1}H NMR spectrum of (±)-nematophin (**NEP-1**) isolated from *X. nematophila* YL001.

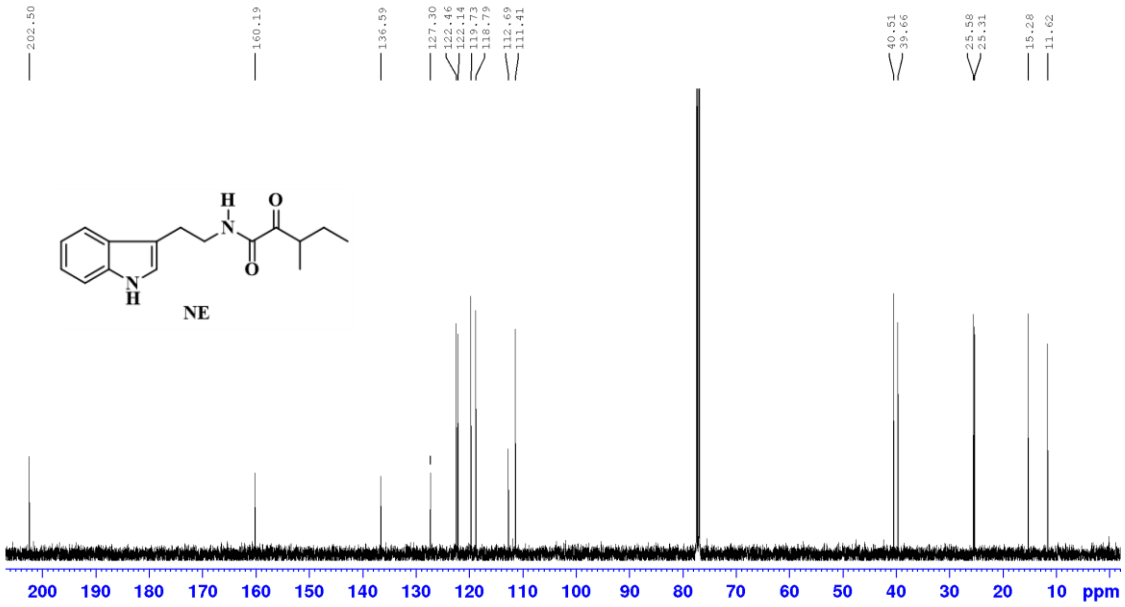

Figure S6 ^{13}C NMR spectrum of (±)-nematophin (**NEP-1**) isolated from *X. nematophila* YL001.

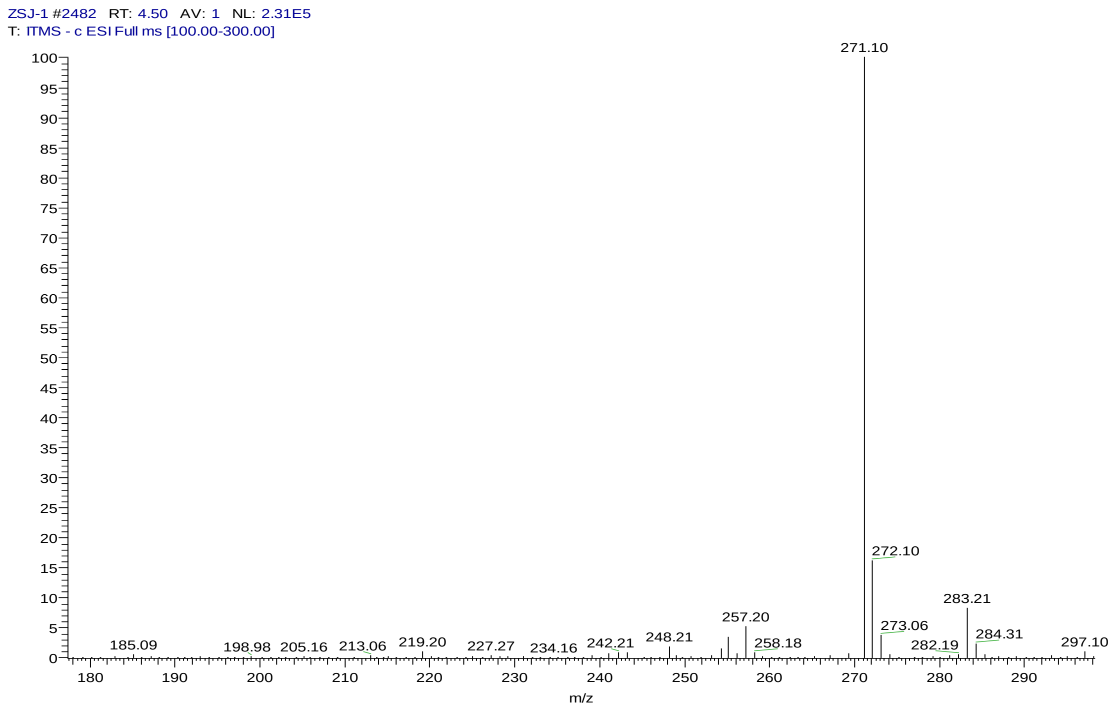

Figure S7 ESI-MS spectrum of (±)-nematophin (**NEP-1**) isolated from *X. nematophila* YL001.

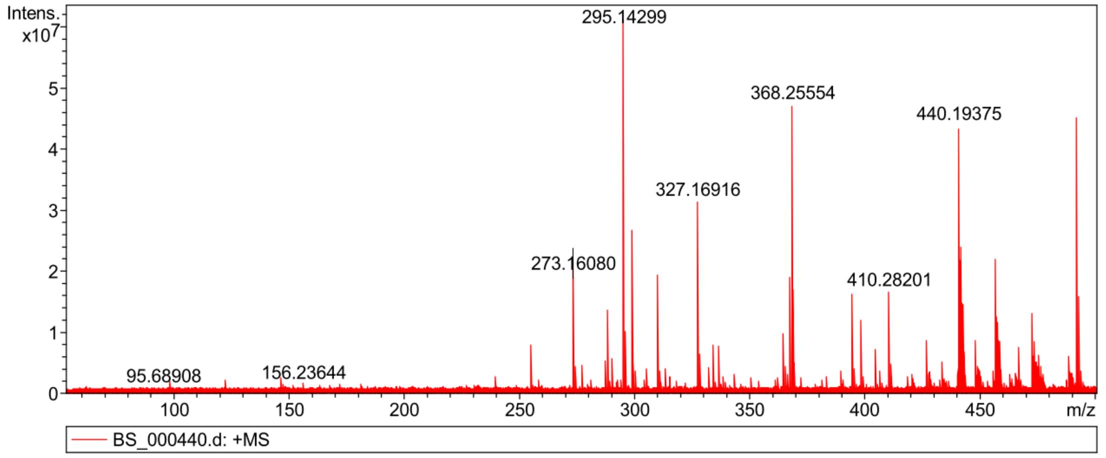

Figure S8 HRESI-MS spectrum of (±)-nematophin (**NEP-1**) isolated from *X. nematophila* YL001.

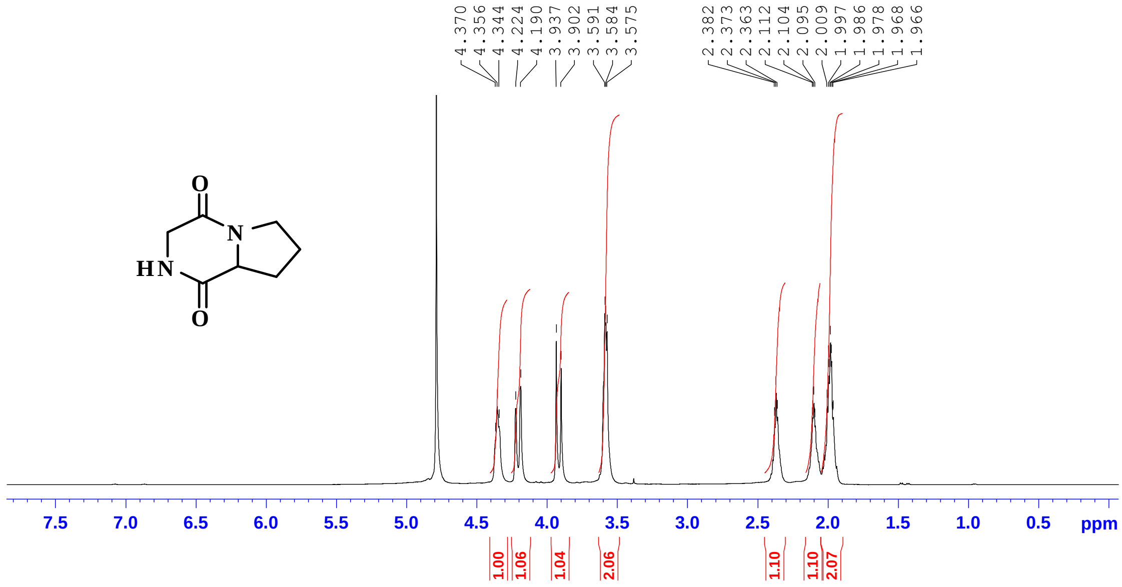

Figure S9 ^{1}H NMR spectrum of *cyclo* (L-Pro-Gly) (**2**).

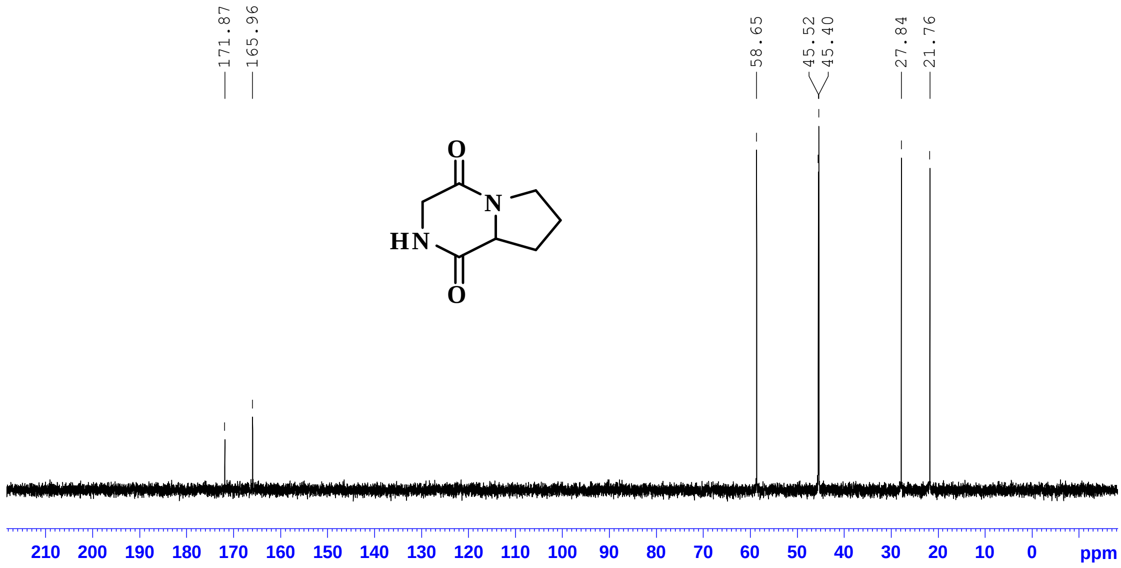

Figure S10 ^{13}C NMR spectrum of *cyclo* (L-Pro-Gly) (**2**).

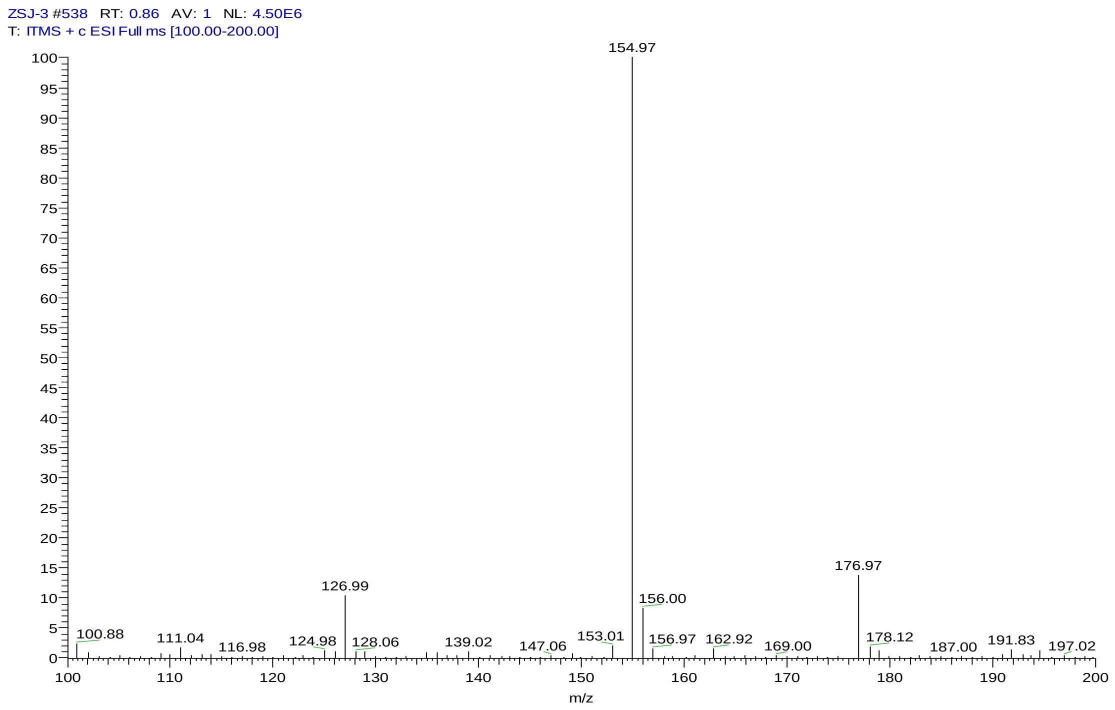

Figure S11 ESI-MS spectrum of *cyclo* (L-Pro-Gly) (**2**).

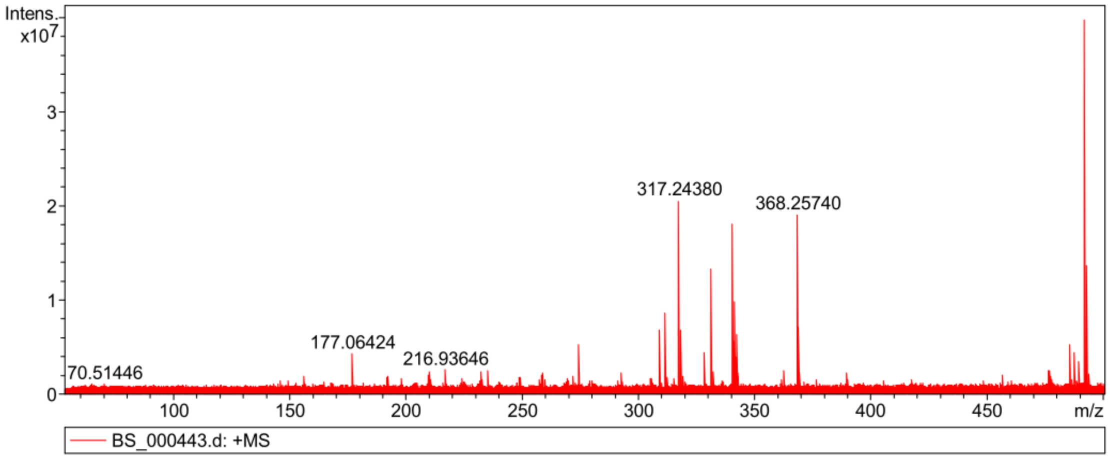

Figure S12 HRESI-MS spectrum of *cyclo* (L-Pro-Gly) (**2**).

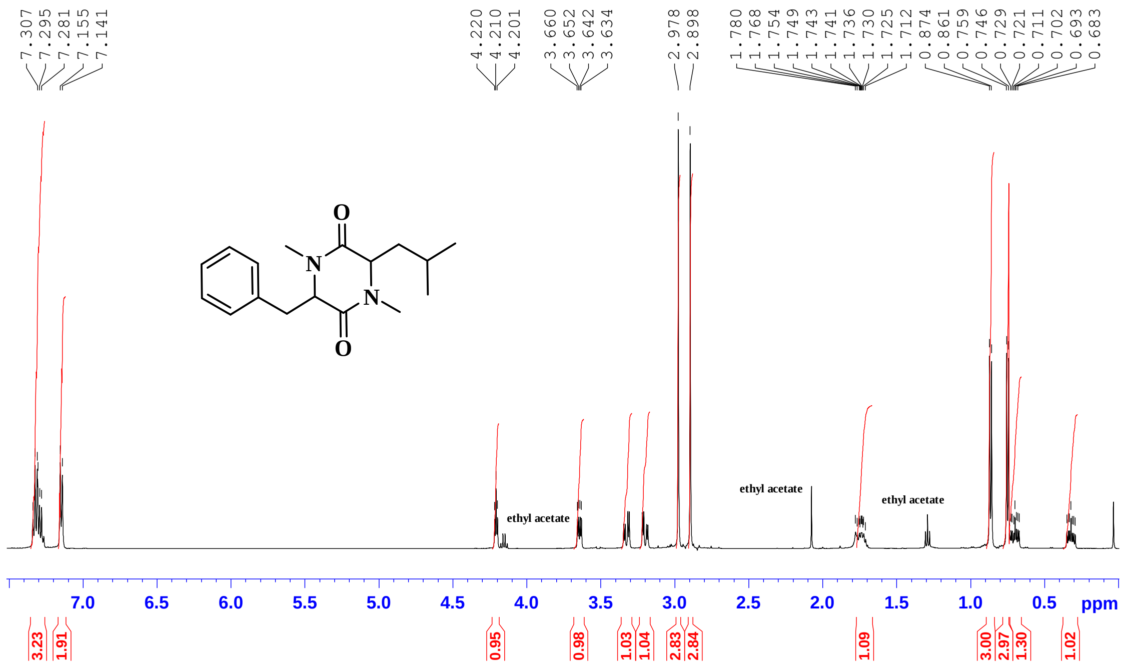

Figure S13 ^{1}H NMR spectrum of N, N’-dimethyl-*cyclo*(L-Phe-L-Leu) (**3**).

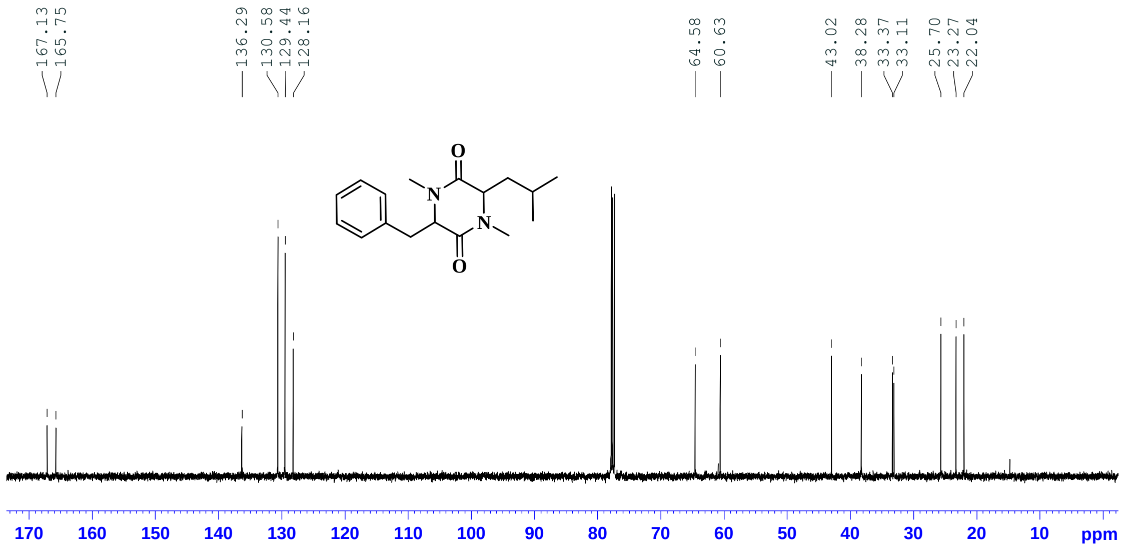

Figure S14 ^{13}C NMR spectrum of N, N’-dimethyl-*cyclo*(L-Phe-L-Leu) (**3**).

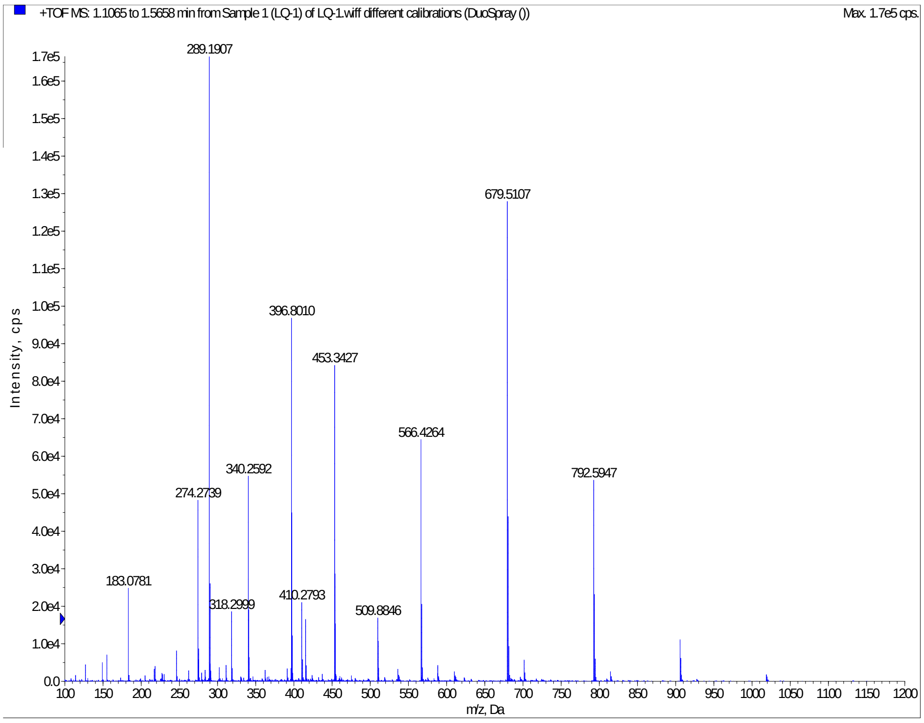

Figure S15 HRESI-MS spectrum of N, N'-dimethyl-*cyclo* (L-Phe-L-Leu) (**3**)

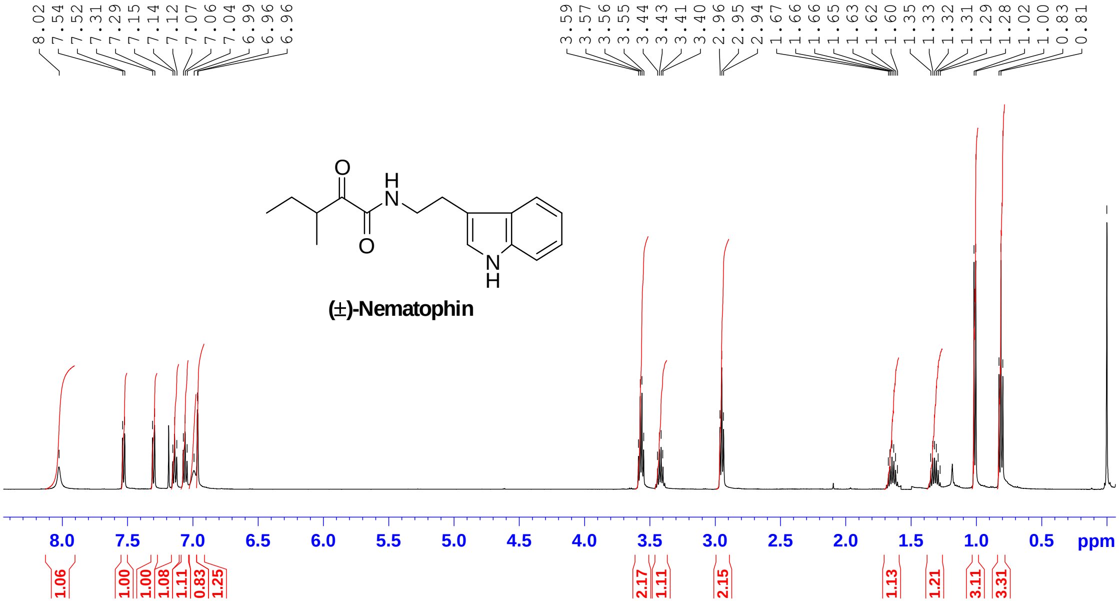

Figure S16 ^{1}H NMR spectrum of synthetic (±)-nematophin (**NEP-2**).

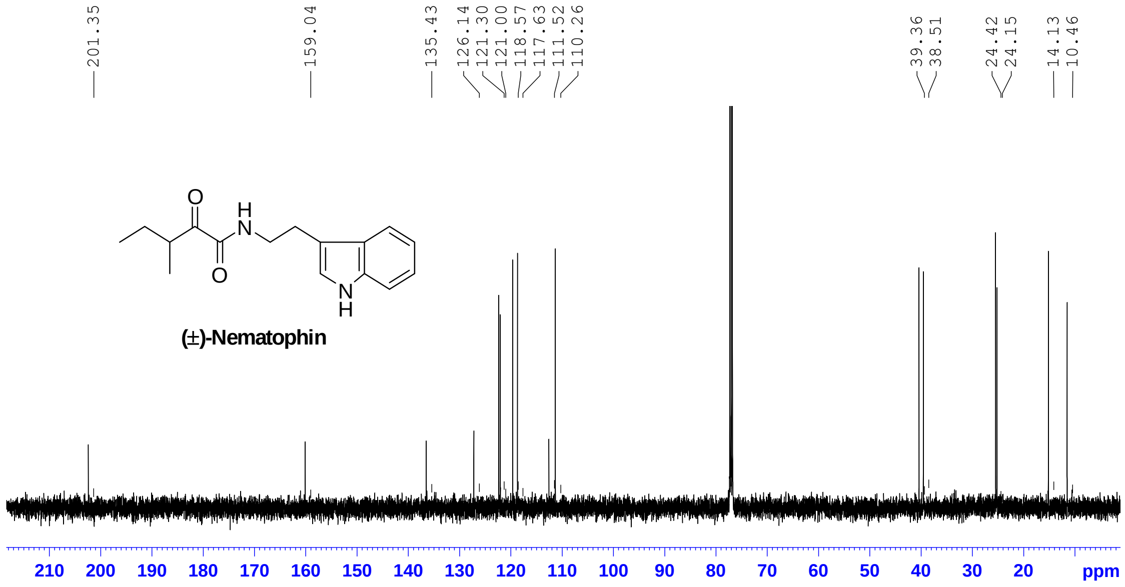

Figure S17 ^{13}C NMR spectrum of synthetic (±)-nematophin (**NEP-2**).

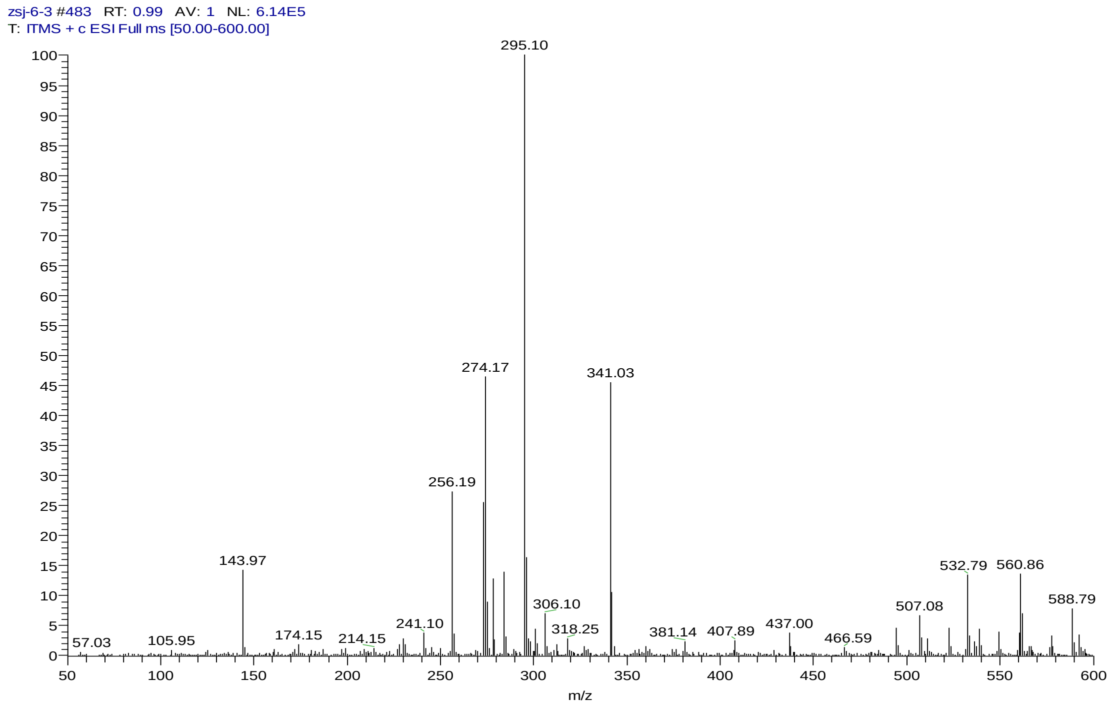

Figure S18 ESI-MS spectrum of synthetic (±)-nematophin (**NEP-2**) in positive mode.

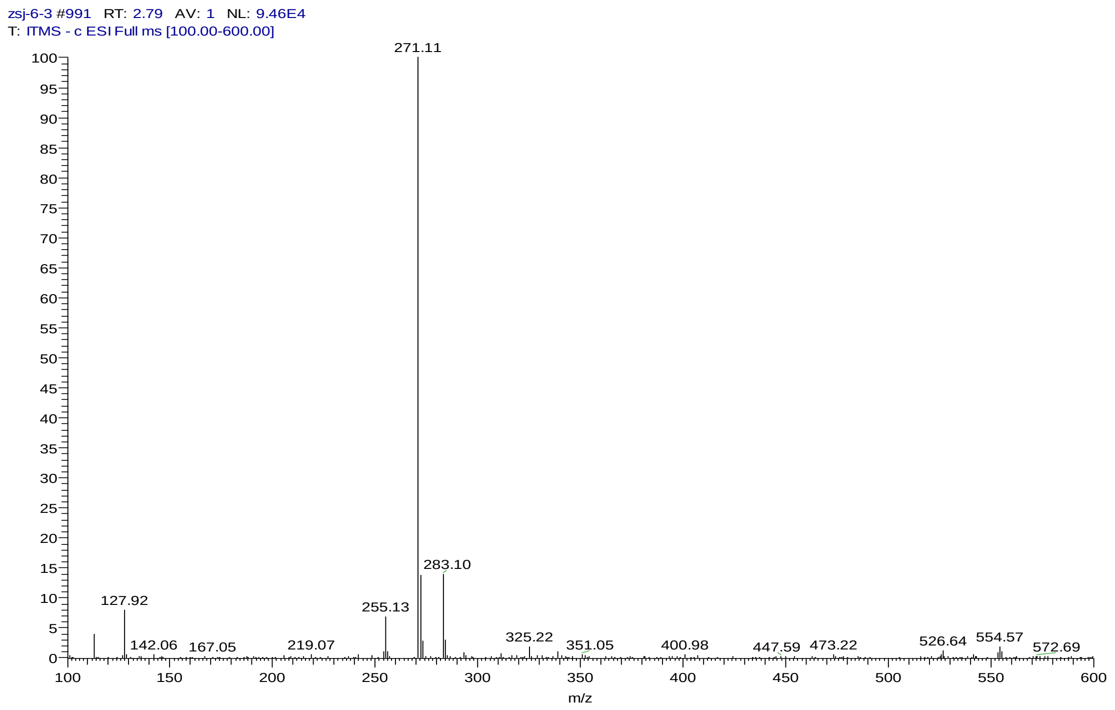

Figure S19 ESI-MS spectrum of synthetic (±)-nematophin (**NEP-2**) in negative mode.

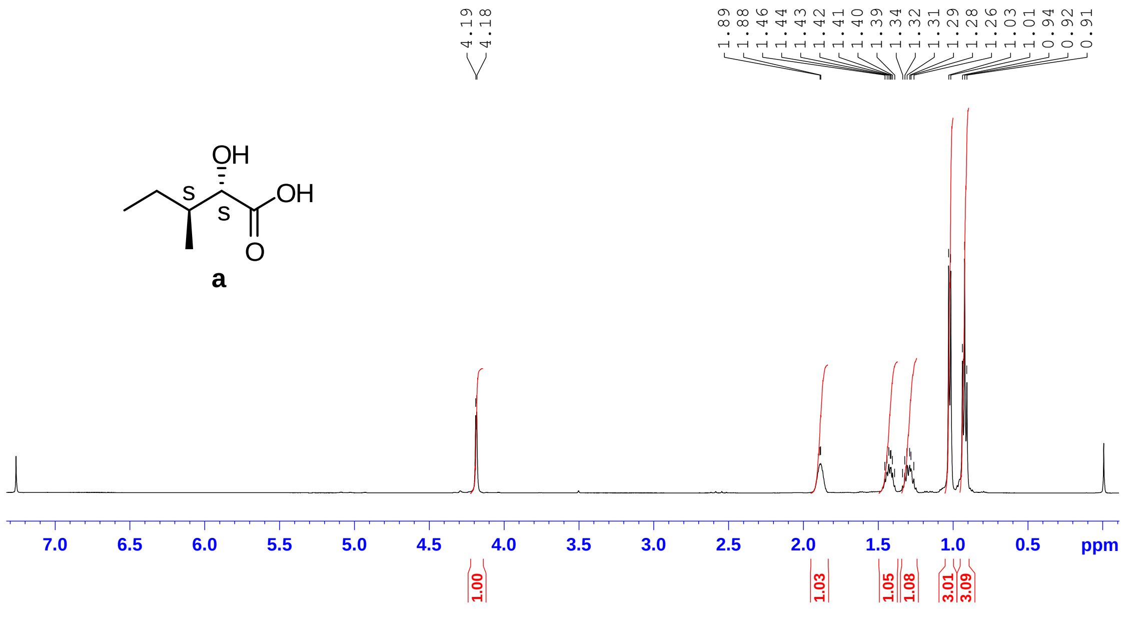

Figure S20 ^{1}H NMR spectrum of (2S,3S)-hydroxypentanoic acid (**a**).

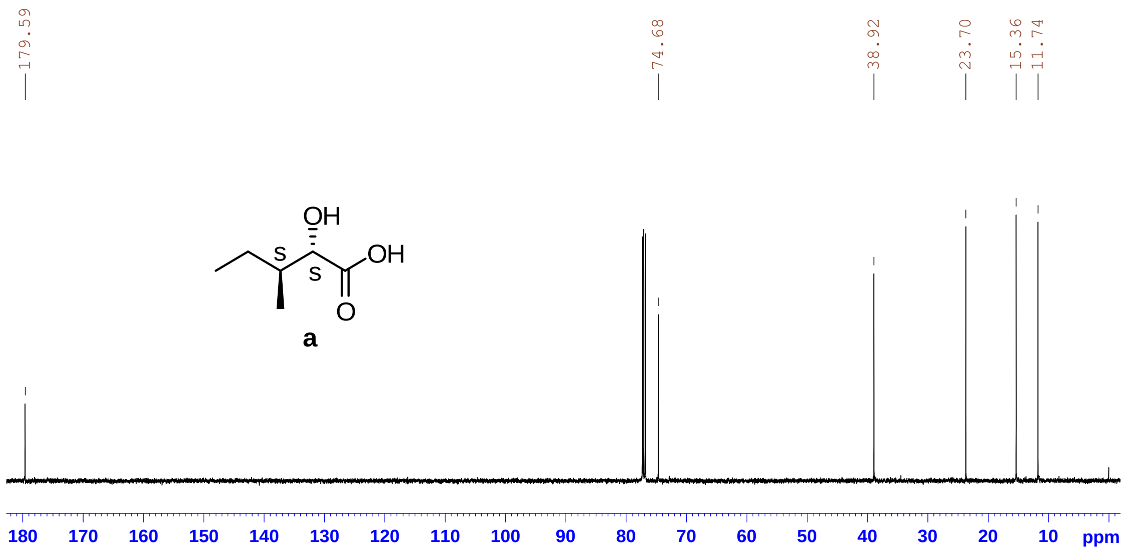

Figure S21 ^{13}C NMR spectrum of (2S,3S)-hydroxypentanoic acid (**a**).

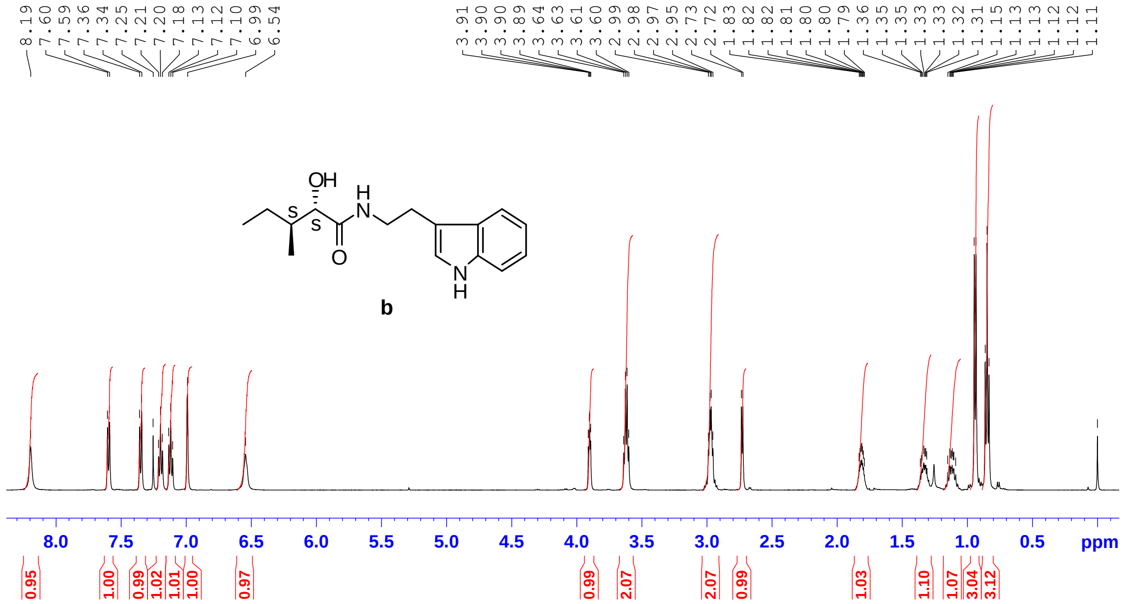

Figure S22 ^{1}H NMR spectrum of (2'S, 3'S)-N-(Indol-3-ylethyl)-2'-hydroxy-3'-methyl pentanamide (**b**).

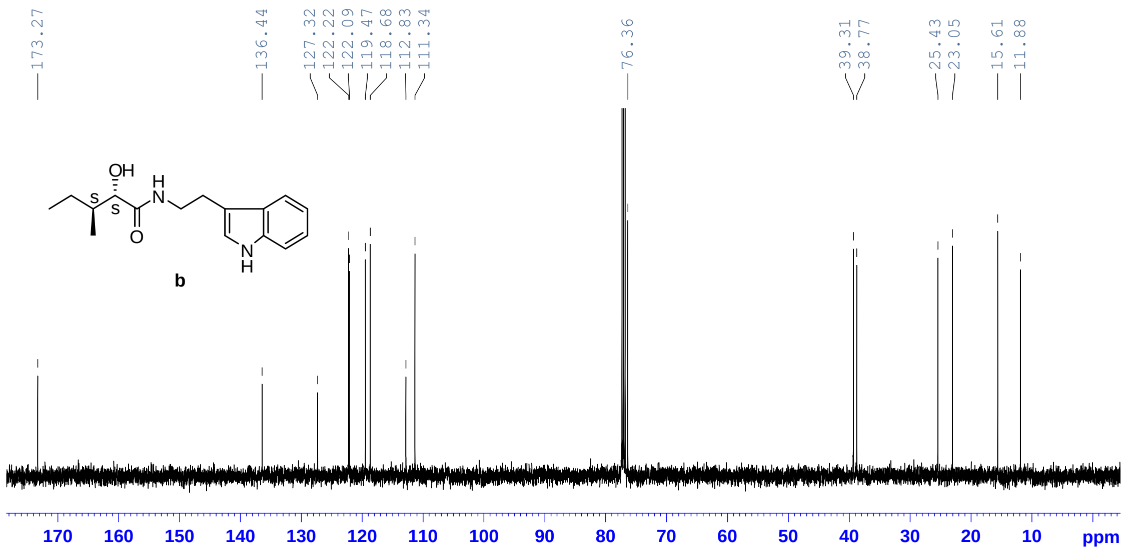

Figure S23 ^{13}C NMR spectrum of (2'S, 3'S)-N-(Indol-3-ylethyl)-2'-hydroxy-3'-methyl pentanamide (**b**).

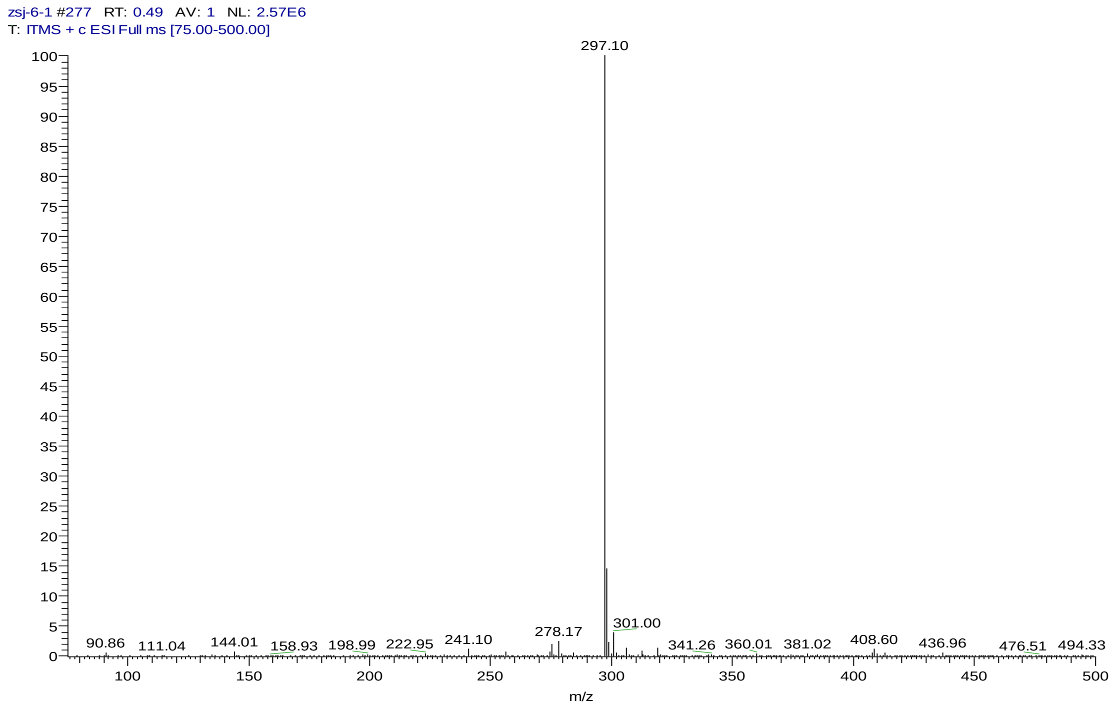

Figure S24 ESI-MS spectrum of (2'S, 3'S)-N-(Indol-3-ylethyl)-2'-hydroxy-3'-methyl pentanamide (**b**).

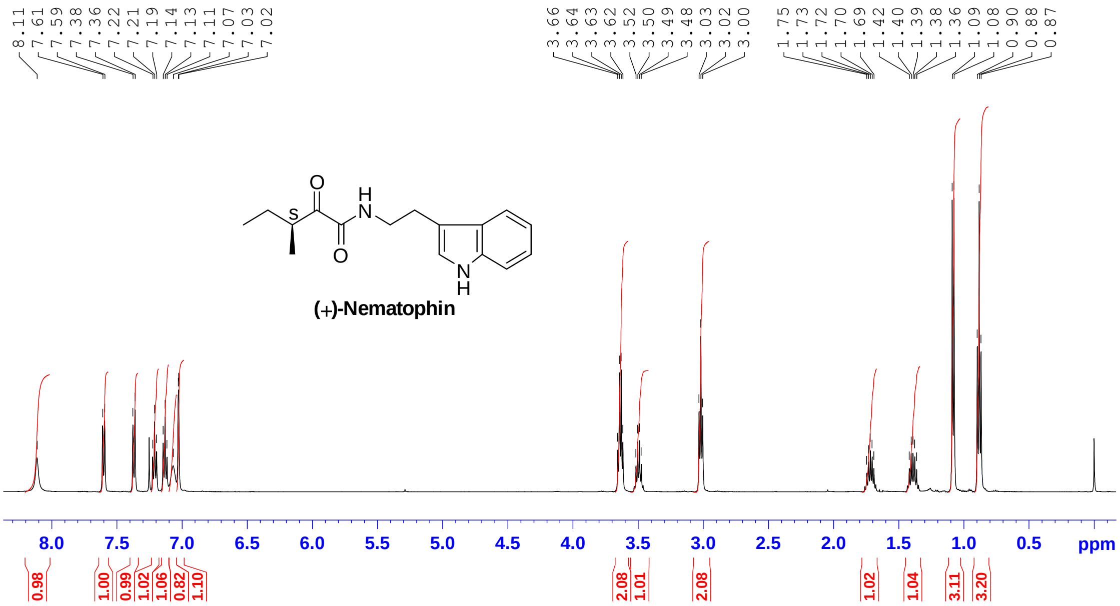

Figure S25 ^{1}H NMR spectrum of synthetic (+)-nematophin (**NEP-3**).

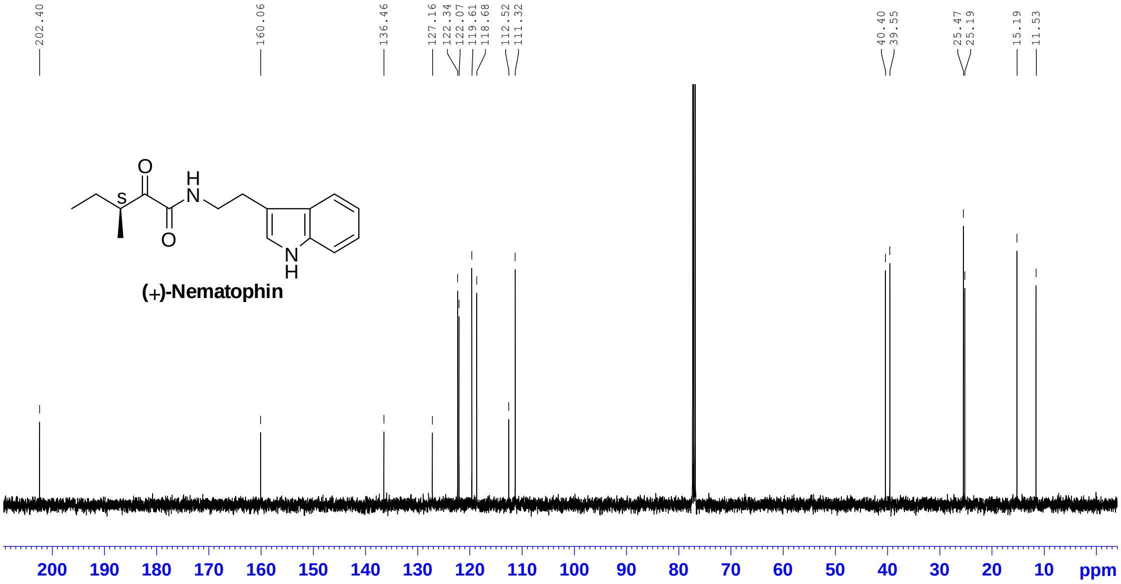

Figure S26 ^{13}C NMR spectrum of synthetic (+)-nematophin (**NEP-3**).

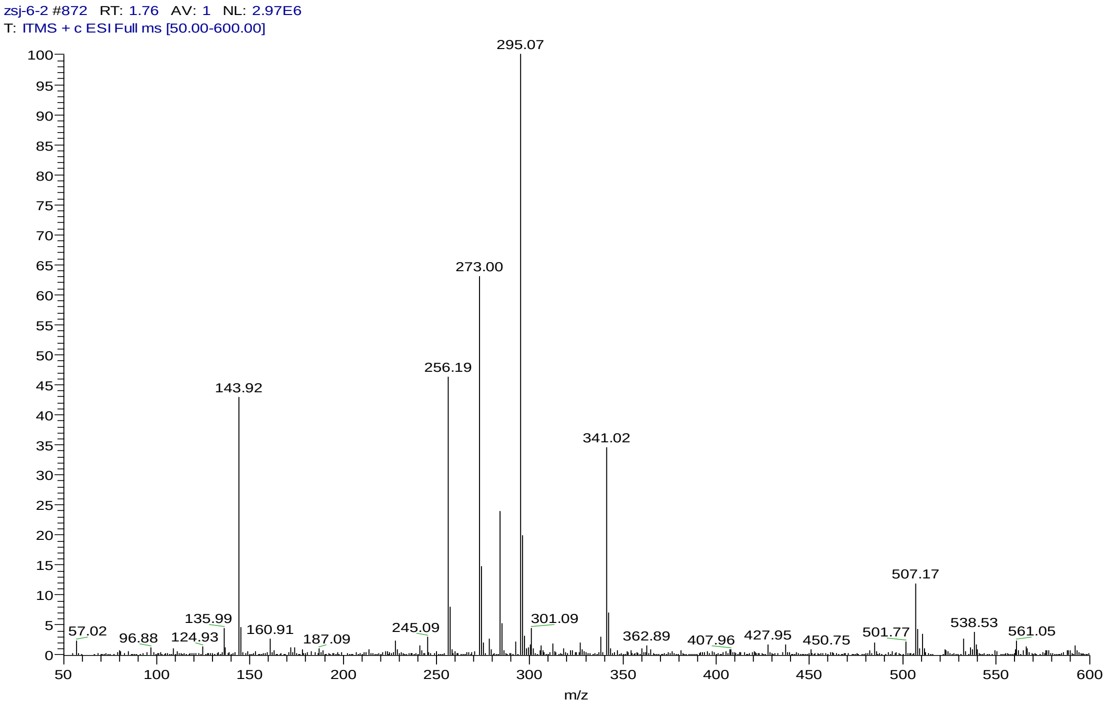

Figure S27 ESI-MS spectrum of synthetic (+)-nematophin (**NEP-3**) in positive mode.

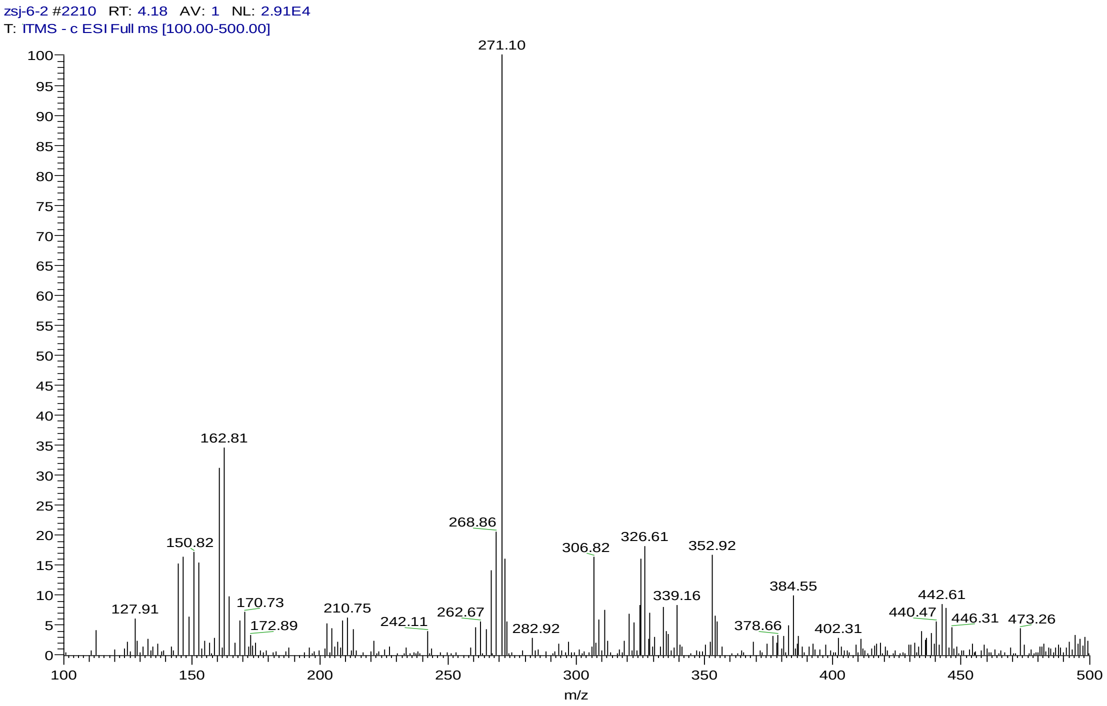

Figure S28 ESI-MS spectrum of synthetic (+)-nematophin (**NEP-3**) in negative mode.

Reference:

Paik, S., Park, M.K., Jhun, S.H., Park, H.K., Lee, C.S., Cho, B.R., et al. (2003). Isolation and Synthesis of Tryptamine Derivatives from a Symbiotic Bacterium *Xenorhabdus nematophilus* PC. *Cheminform* 34(45)**,** 2101-2118. doi: 10.1002/chin.200345209

YH, Wang, X, Zhang (2006). Isolation and preliminary identification of two strains of entomopathogenic nematode symbionts. Jour. of Nor thwest Sci-Tech Univ. of Agri. and For. (Nat. Sci. Ed.), 34(12)**,** 174-180. doi:10.3321/j.issn:1671-9387.2006.12.034.
